# Control of renal central carbon metabolism by heme oxygenase-1

**DOI:** 10.1016/j.isci.2025.114370

**Published:** 2025-12-06

**Authors:** Joel Guerra, Elisa Jentho, Sascha Schäuble, Brendon P. Scicluna, Wolfgang Vivas, Dania Martínez-Alarcón, Marco Groth, Emma King, Franziska Röstel, Gianna Hirth, Miguel P. Soares, Thorsten Wiech, Gianni Panagiotou, Michael Bauer, Verena Hoerr, Sebastian Weis

**Affiliations:** 1 Department of Anesthesiology and Intensive Care Medicine, Jena University Hospital, Friedrich-Schiller University, Jena, Germany; 2 Center for Sepsis Control and Care, Jena University Hospital, Friedrich-Schiller University, Jena, Germany; 3 Gulbenkian Institute for Molecular Medicine, Lisboa, Portugal; 4 Institute of Pathology, University Medical Center Hamburg-Eppendorf, Hamburg, Germany; 5 Department of Microbiome Dynamics, Leibniz Institute for Natural Product Research and Infection Biology - Hans-Knöll Institute (Leibniz-HKI), Jena, Germany; 6 Department of Applied Biomedical Science, Faculty of Health Sciences, Mater Dei Hospital, University of Malta, Msida, Malta; 7 Centre for Molecular Medicine and Biobanking, Biomedical Sciences, University of Malta, Msida, Malta; 8 Institute for Infectious Disease and Infection Control, Jena University Hospital, Friedrich-Schiller University, Jena, Germany; 9 Leibniz Institute for Natural Product Research and Infection Biology, Hans-Knöll Institute- HKI, Jena, Germany; 10 Genome Analysis, Leibniz Institute on Aging - Fritz Lipmann Institute (FLI), Jena, Germany; 11 Department of Medicine and State Key Laboratory of Pharmaceutical Biotechnology, University of Hong Kong, Hong Kong, China; 12 Friedrich Schiller University, Faculty of Biological Sciences, Jena, Germany; 13 Institute of Medical Microbiology, Jena University Hospital, Friedrich Schiller University Jena, Jena, Germany; 14 Heart Center Bonn, Department of Internal Medicine II, University Hospital Bonn, Bonn, Germany

**Keywords:** Molecular physiology, Cell biology

## Abstract

Metabolic adaptation is an integral part of the organismal stress-response. In this study, we investigate the role of Hmox1 in mediating metabolic adaptation under both physiological and hemolytic stress conditions. Using an inducible *Hmox1* deletion model (*Hmox1*^*R26Δ/Δ*^), we demonstrate that *Hmox1* expression is essential for preventing heme-induced kidney failure. Our integrative approach, combining bulk RNA sequencing and targeted metabolomics within silico metabolic modeling, revealed a compromised pentose phosphate pathway (PPP) in failed renal adaptation after *Hmox1* deletion. This study underscores the critical role of *Hmox1* for PPP regulation and sheds light on the metabolic pathways involved in kidney dysfunction due to hemolytic diseases.

## Highlights

• Conditional deletion of *Hmox1* does not impair vigor nor does it reduce lifespan in mice
• *Hmox1* expression is vital for averting heme-induced acute kidney failure
• *Hmox1* modulates metabolic stress adaptation via the pentose phosphate pathway
• The gene mediates a kidney-specific protective role

## Graphical abstract

**Figure:**
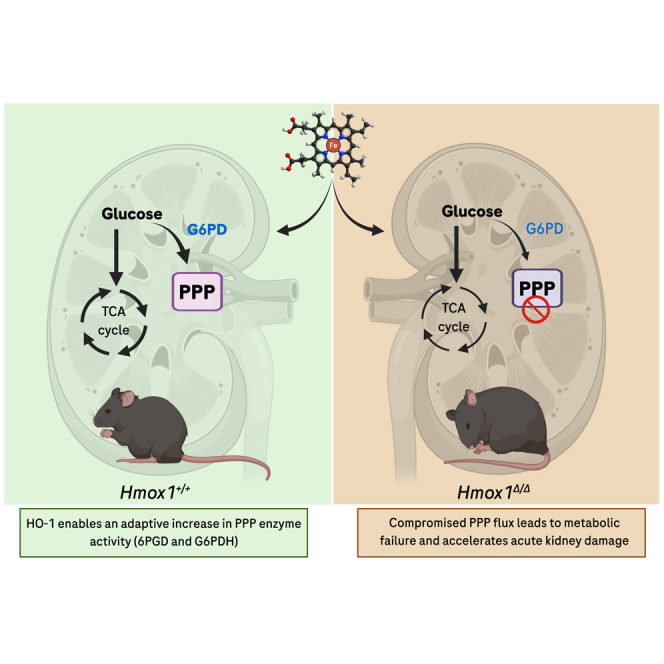

## Introduction

Adaptation of cellular metabolism is a fundamental defense mechanism against diverse stresses posed by environmental factors and infectious agents.^1^^,^^2^ Changes in specific metabolic pathways directly relate to the progression and severity of communicable and non-communicable diseases.^3^^,^^4^^,^^5^^,^^6^ This metabolic “rewiring” is instrumental in modulating, e.g., immune responses and bridging the gap between cellular metabolism and the function of innate and adaptive immunity.^1^^,^^7^

Heme, an evolutionarily conserved iron-containing protoporphyrin acts as an indispensable prosthetic group for hemoproteins and mainly serves for oxygen transportation.^8^^,^^9^ Labile heme, i.e., cell-free heme, is a redox-active molecule that oxidizes lipids and proteins and can cause DNA damage promoting cytotoxicity and tissue damage.^8^^,^^9^ This is critical in the pathogenesis of diseases such as malaria^10^ and bacterial sepsis.^9^^,^^11^ Labile heme also acts as an alarmin^12^ by signaling via Toll-like receptor-4^13^ and the C-type lectin-like receptor-2,^14^ and can activate the pyrin domain containing (NLRP) 3 inflammasome.^15^

To counter the potential damage from labile heme, mammals have evolved two distinct heme oxygenases: the inducible isoform HO-1 encoded by *HMOX1* and the constitutively expressed isoform HO-2, encoded by *HMOX2*.^16^ Induction of HO-1 has been widely associated with beneficial effects against acute and chronic inflammation.^17^^,^^18^^,^^19^^,^^20^ *Hmox1* expression is generally cytoprotective due to (1) the removal of labile heme; (2) generation of antioxidant and signaling activities of the byproducts of heme catabolism, namely, carbon monoxide, biliverdin, and ferrous iron; and (3) direct enzymatic-independent activation of gene transcription.^8^^,^^21^ The exploration of its role in disease mitigation and metabolic adaptation has been constrained by methodological limitations in previous studies, which relied on *Hmox1*-deficient mouse models with high embryonic lethality. The few surviving *Hmox1*^−/−^ mice exhibited increased infection mortality rates. Tissue-specific, *Hmox1* deletions are not deleterious in mice^19^^,^^22^^,^^23^^,^^24^ but cannot fully elucidate the systemic functions of the gene^17^^,^^25^ since tissues that have not been deleted may continue to affect whole-body heme and iron homeostasis.^26^

We here employ conditionally *Hmox1*-deficient mice (*Hmox1*^*R26Δ/Δ*^), which allows us to bypass the limitations of previous models and directly assess the consequences of induced *Hmox1* deletion on systemic stress responses to heme.^27^

## Results

### Conditional deletion of *Hmox1* impairs tissue damage control to labile heme

**Figure 1:**
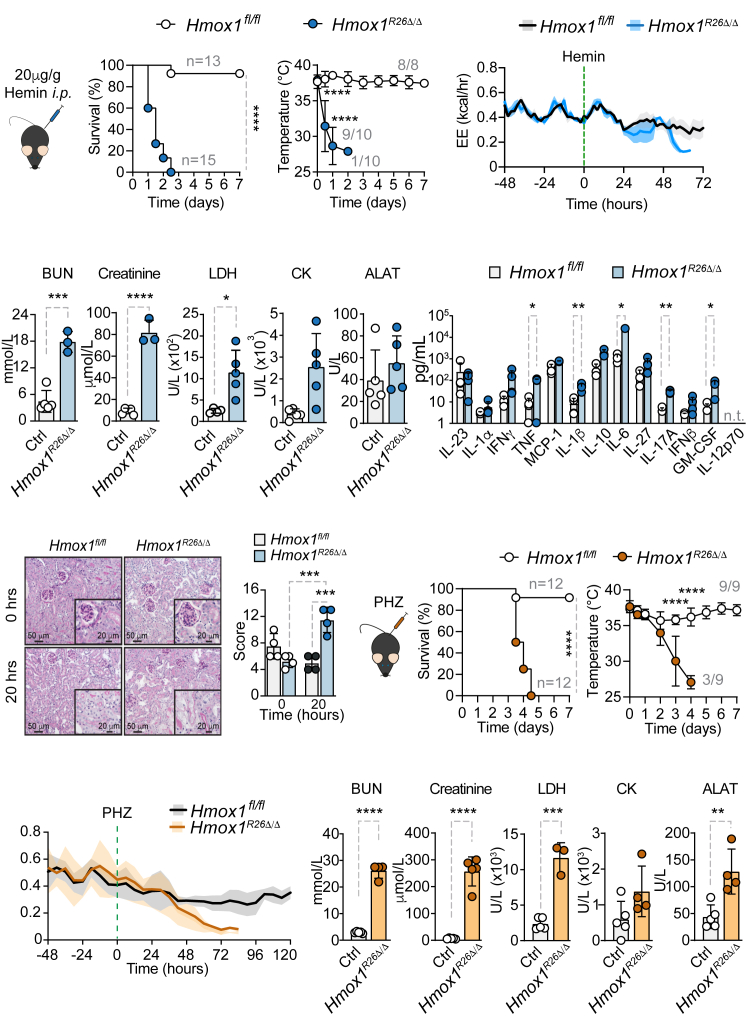
*Conditional Hmox1 deletion* impairs tissue damage control to heme-mediated stress (A) Survival, (B) temperature, and (C) energy expenditure of *Hmox1*^*fl/fl*^ (Ctrl) and *Hmox1*^*R26Δ/Δ*^ animals subjected to 20 mg/kg heme *i.p*. (D) Organ dysfunction markers 20 h and (E) plasma cytokines measured by Legendplex 12 h after heme application. (F) PAS staining of kidney from *Hmox1*^*fl/fl*^ (Ctrl) and individual *Hmox1*^*R26Δ/Δ*^ animals. Representative of four different animals. (G) Scoring of kidney damage. (H) Survival, (I) temperature, and (J) energy expenditure of *Hmox1*^*fl/fl*^ (Ctrl) and individual *Hmox1*^*R26Δ/Δ*^ animals subjected to PHZ. (K) Organ damage markers 72 h after PHZ application. *n* = 3 independent experiments. Gray numbers indicate animal numbers. Dots represent individual animals. All data are presented as mean ± SD. ∗*p* < 0.05, ∗∗*p* < 0.01, ∗∗∗*p* < 0.001, ∗∗∗∗*p* < 0.0001. Student’s *t* test was used for comparison between two groups. ALAT, alanine aminotransferase; BUN, blood urea nitrogen; CK, creatine kinase; Ctrl, control (*Hmox1*^*fl/fl*^); LDH, lactate dehydrogenase; PHZ, phenylhydrazine.

We have previously shown that labile heme induces sickness behavior and a transient decrease in energy expenditure.^28^ In this study, we sought to investigate the effects of heme in tamoxifen-induced ubiquitous *Hmox1* (*Hmox1*^*R26Δ/Δ*^) deleted animals. *Hmox1*^*R26Δ/Δ*^ mice that received 20 mg/kg hemin intraperitoneal (*i.p*.) succumbed rapidly in contrast to control animals with a physiological expression of *Hmox1* (*Hmox1*^*fl/fl*^) (Figure 1A). *Hmox1*^*R26Δ/Δ*^ mice, but not the control group, developed severe hypothermia and hypoglycemia (Figures 1B, S1D, and S1E) in conjunction with energetic failure, as indicated by decreased energy expenditure (Figure 1C), oxygen consumption (VO_2_), and activity (Figures 1B, S1G, and S1H). At steady state, energy expenditure did not differ between both genotypes (Figures 1C and S1G). Male and female animals had the same phenotype. Serology analysis revealed the development of acute kidney failure in *Hmox1*^*R26Δ/Δ*^ mice, but no evidence of liver dysfunction after hemin injection (Figure 1D). Since heme acts as an alarmin,^13^^,^^14^ we assessed cytokine release in plasma and the peritoneal cavity. In agreement with previous studies,^18^^,^^29^ heme promoted an inflammatory phenotype in *Hmox1*^*R26Δ/Δ*^ animals, which exhibit significantly increased cytokine release, compared to *Hmox1*^*fl/fl*^ controls (Figures 1E and S1F). Histological examination revealed enhanced tubular dilatation, peritubular capillaries (PTC) dilatation, epithelial flattening, brush border loss, and vacuolization in *Hmox1*^*R26Δ/Δ*^ vs. *Hmox1*^*fl/fl*^ animals 20 h after hemin injection, indicative of direct kidney damage (Figure 1F). Next, we subjected mice to phenylhydrazine (PHZ), a chemical that causes intravascular hemolysis, serving as a model of sterile hemolytic stress.^30^ The results corroborated our previous observations, showing enhanced mortality, hypothermia, energetic collapse, acute kidney injury, and additional weight loss in *Hmox1*^*R26Δ/Δ*^ but not in *Hmox1*^*fl/fl*^ (Figures 1G–1J and S1I–S1L). This supports the notion that *Hmox1* fundamentally governs energy homeostasis in response to heme exposure, potentially underlying hemolysis-induced acute renal failure.^27^^,^^31^^,^^32^ Constitutive deletion of *Hmox1* is known to be predominantly lethal at the embryonic stage. The few surviving *Hmox1*^−/−^ animals display an inflammatory phenotype characterized by kidney dysfunction, premature death, splenomegaly, and tissue iron deposition.^17^^,^^25^ However, *Hmox1*^*R26Δ/Δ*^ animals did not exhibit any of these deleterious traits (Figures S2A*–*S2F) up to 12 months after deletion and presented with unchanged gluconeogenesis capacity (Figures S3A*–*S3D). As such, we conclude that *Hmox1* expression does not critically influence organismal homeostasis, under physiological conditions. Nonetheless, conditional deletion of *Hmox1* impairs tissue damage control to labile heme.

### Heme-induced mortality in *Hmox1*-deficient mice cannot be prevented

**Figure 2:**
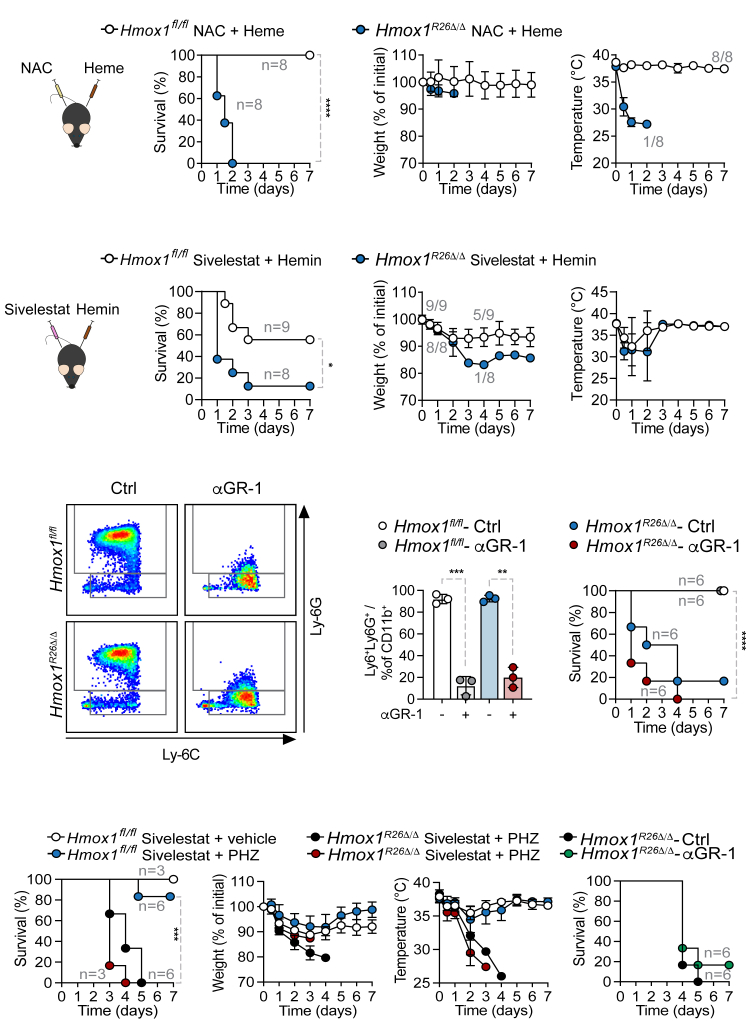
Heme lethality is immune system and redox status independent (A) Survival, (B) relative weight, and (C) temperature of *Hmox1*^*fl/fl*^ and *Hmox1*^*R26Δ/Δ*^ mice injected *i.p*. with NAC 1 h prior to 20 mg/kg hemin injection, and once a day thereafter. (D) Survival, (E) relative weight, (F) temperature of *Hmox1*^*fl/fl*^ and *Hmox1*^*R26Δ/Δ*^ mice injected *i.p*. with sivelestat 1 h prior to 20 mg/kg heme injection, and once a day thereafter. Two independent experiments, *n* = 6 animals per group. (G) Gating strategy, (H) flow cytometry analysis, and (I) survival of *Hmox1*^*fl/fl*^*and Hmox1*^*R26Δ/Δ*^ mice injected *i.p.* with anti-GR1 antibody or isotype 24 h prior to 20 mg/kg heme injection, and once every 3 days thereafter. Two independent experiments, *n* = 6 animals per group. (J) Survival, (K) relative weight, (L) temperature of *Hmox1*^*fl/fl*^ and *Hmox1*^*R26Δ/Δ*^ mice injected *i.p*. with sivelestat 1 h prior to PHZ injection. Two independent experiments, *n* = 3–6 animals per group. (M) Survival of *Hmox1*^*R26Δ/Δ*^ mice injected *i.p.* with anti-GR1 antibody or isotype 24 h prior to PHZ injection. Data derived from two independent experiments. All data are presented as mean ± SD. Flow cytometry data were analyzed with multiple unpaired Student’s *t* test with Welch correction. ∗*p* < 0.05, ∗∗*p* < 0.01, ∗∗∗*p* < 0.001, ∗∗∗∗*p* < 0.0001. Ctrl, control (*Hmox1*^*fl/fl*^); NAC, N-acetyl cysteine; PHZ, phenylhydrazine.

Heme can induce kidney injury through the disruption of redox homeostasis and lipid peroxidation.^8^^,^^33^^,^^34^^,^^35^ Based on this, we aimed to prevent heme-induced mortality in *Hmox1*^*R26Δ/Δ*^ animals. However, N-acetylcysteine (NAC) administration failed to prevent heme-associated lethality in *Hmox1*^*R26Δ/Δ*^ mice (Figures 2A–2C). Given the increased cytokine release in *Hmox1*^*R26Δ/Δ*^ animals (Figures 1E and S1F) and the established link between heme, an exacerbated neutrophil activation and NETosis,^36^^,^^37^^,^^38^^,^^39^ we hypothesized that heme-induced lethality in *Hmox1*^*R26Δ/Δ*^ mice could be attributed to neutrophil activation and infiltration. However, our findings showed that animals treated with sivelestat, an inhibitor of neutrophil infiltration,^40^ could not prevent heme-induced lethality in *Hmox1*^*R26Δ/Δ*^ mice (Figures 2D–2F). Since expression of *Hmox1* in myeloid cells has been shown to improve kidney damage in ischemia-reperfusion and hemolysis-induced lethality, which involves inflammasome activation by heme,^30^^,^^41^ we next mitigated the impact of the immune response via depleting GR-1^+^ cells (Figures 2G and 2H). Also, this did not impact heme-induced lethality in *Hmox1*^*R26Δ/Δ*^ mice (Figure 2I). The same was observed when using PHZ (Figures 2J*–*2M). We ultimately manipulated glucose homeostasis as it had previously been shown that stress-associated hypoglycemia can be reversed with glucose administration resulting in enhanced survival of mice.^4^ This approach however could not prevent heme-induced lethality in *Hmox1*^*R26Δ/Δ*^ mice (Figures S4A*–*S4D). The cytotoxic effects appear to be mediated by heme directly, since the application of iron (Figures S4E*–*S4H) did not reproduce the phenotype nor did the application of ferrostatin, an inhibitor of an iron-dependent cell death mechanism known as ferroptosis^42^^,^^43^ (Figures S4I–S4L) prevents heme-induced mortality in *Hmox1*^*R26Δ/Δ*^ animals. Furthermore, we assessed whether the sole expression of *Hmox1* in the proximal kidney epithelial cells account for the observed protection against heme-mediated metabolic failure. Neither hemin nor PHZ application in animals with a specific deletion of *Hmox1* in proximal kidney epithelial cells (*Hmox1*^*PepckΔ/Δ*^)^22^ resulted in relevant mortality or altered disease severity when compared to control *Hmox1*^*fl/fl*^ animals (Figures S4M and S4N).

### Expression of *Hmox1* induces distinct transcripts that maintain kidney function

**Figure 3:**
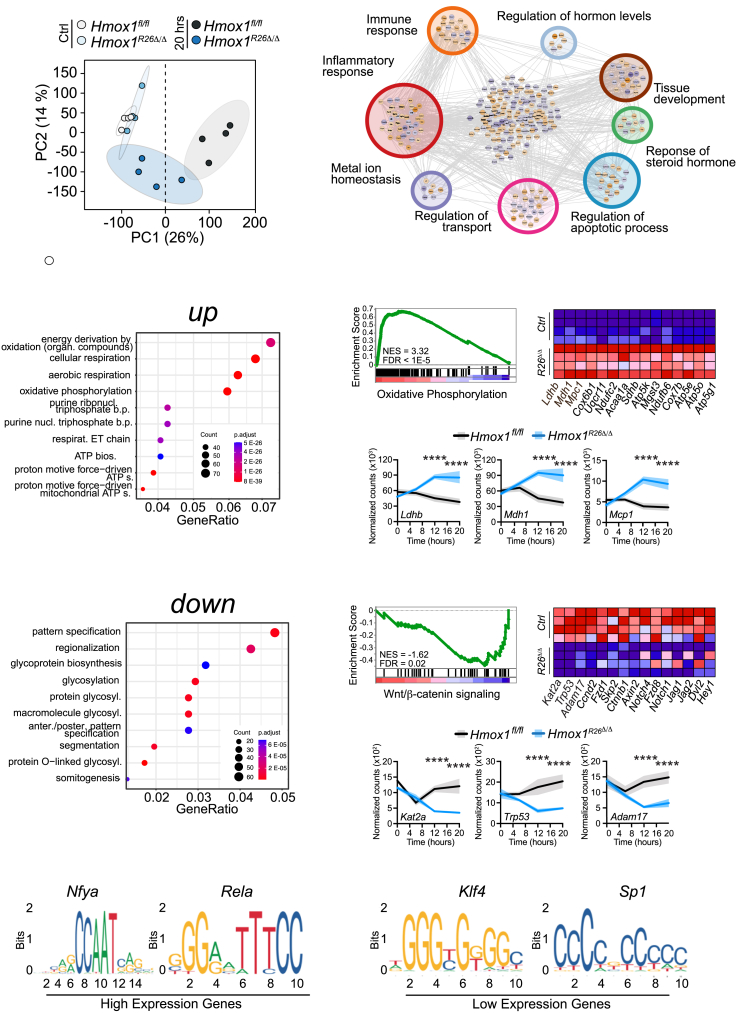
*Hmox1* expression induces distinct transcripts protecting kidney function (A) Principal-component analysis (PCA) plot. (B) STRING network analysis on RNA sequencing data for the genes differentially expressed 20 h after heme application. (C) Gene ontology: biological process (GOBP) 10 most upregulated pathways. (D) Barcode plot highlighting the upregulated genes of oxidative phosphorylation in *Hmox1*^*R26Δ/Δ*^ vs. *Hmox1*^*fl/fl*^ animals, (E) heatmap showing lead 15 upregulated genes from (D). (F) Time-resolved mRNA expression of three first genes identified in the GSEA. Data are derived from individual mice per time point but displayed with a connecting line for better visualization. (G) Ten most downregulated pathways using GoBP. (H) GSEA plot highlighting the down-regulated genes of Wnt/β-catenin synthesis pathways in *Hmox1*^*R26Δ/Δ*^ vs. *Hmox1*^*fl/fl*^ animals. (I) Heatmap showing lead 15 downregulated genes from (H). (J) Time-resolved mRNA expression of the three first genes identified in the GSEA. Data are derived from individual mice per time point but displayed with a connecting line for better visualization. (K and L) Promoter sequence analysis revealing significantly enrichment for *Nfya* and *relA* transcription factor-binding sites in upregulated genes and enrichment of Klf4 and Sp1 transcription factor binding site for genes found to be down-regulated. *n* = 4 per group. Data are shown as mean +SD from 3 to 4 animals per group. two-way ANOVA with Šidák correction: ∗*p* < 0.05; ∗∗*p* < 0.01; ∗∗∗*p* < 0.001; ∗∗∗∗*p* < 0.0001.

Having established that *Hmox1*^*R26Δ/Δ*^ mice succumb to acute renal failure following heme exposure, we conducted a time series bulk RNA sequencing analysis of kidneys from *Hmox1*^*R26Δ/Δ*^ and control animals (Figure 3) at baseline, 6, 12, and 20 h post hemin application. Transcriptomic profiling revealed several baseline differences (Figures S5A*–*S5C), which were not correlated with vigor, organ damage or cytokine release under physiological conditions (Figure S3). Principal component analysis (PCA) demonstrated clustering of hemin-treated *Hmox1*^*R26Δ/Δ*^ animals (Figure 3A). STRING-DB network analysis unveiled various differentially activated biological processes involved in the immune response, metabolic processes, and repair (Figure 3B). We then conducted a comparative analysis between *Hmox1*^*R26Δ/Δ*^ and littermate controls at each time point to identify enriched pathways. Gene set enrichment analysis (GSEA) revealed an upregulation of pathways associated with energy metabolism and ATP production (Figure 3C). This observation was further confirmed in the hallmark gene set through GSEA, where the expression of genes related to oxidative phosphorylation exhibited the highest enrichment (*Hmox1*^*R26Δ/Δ*^ vs. *Hmox1*^*fl/fl*^ NES 3.32; false discovery rate [FDR] <10^−5^) (Figures 3D–3F and S5C). In contrast, pathways related to pattern recognition, biosynthesis, and glycosylation were downregulated (Figure 3G). Additionally, a significant decrease was noted in the wingless-related integration site (Wnt)/β-catenin signaling pathway (NES-1.61; FDR = 0.02) (Figures 3H–3J). At baseline, we observed a downregulation of fatty acid metabolism (NES -1.61; FDR = 0.018) and oxidative phosphorylation (NES-2.76; FDR = FDR <10^−5^), coupled with an upregulation of the Wnt/β-catenin signaling pathway in *Hmox1*^*R26Δ/Δ*^ animals (NES-2.76; FDR = FDR<10^−5^). However, 20 h after hemin application, these pathways were reversed, i.e., fatty acid metabolism was upregulated and Wnt/β-catenin signaling pathway was downregulated (Figure S5C). The influence of *Hmox1* expression on these pathways was further supported by a differential time series enrichment specific to the genotype (Figures S5D–S5G). Bulk-RNA sequencing results were independently verified (Figure S5H). Examining individual gene expression sets, we observed variations in genes related to fatty acid metabolism,^44^ redox homeostasis (deputed to counter lipid peroxidation and the overall stress response), and genes regulating glycolysis, mitochondrial function, and the pentose-phosphate pathway^45^ (PPP) (Figures 3F, 3J, and S6). Promoter sequence analysis unveiled a significant enrichment of *Nuclear transcription factor Y subunit alpha* (*Nfya)* and *relA* transcription factor-binding sites in upregulated genes, while downregulated genes showed an enrichment of the zinc-finger transcription factors *Krüppel-like factor 4* (*Klf4*) and *specificity protein 1* (*Sp1*) (Figure 3K). These findings align with our earlier observations, seeing relA, a component of the nuclear factor κB (NF-κB) transcription factor complex, which is a key regulator of immune and inflammatory responses.^46^ Additionally, Sp1 has been implicated in the regulation of various metabolic processes, including glucose metabolism, and can influence the expression of genes involved in glycolysis, gluconeogenesis, and lipid metabolism.^47^

### Expression of *Hmox1* preserves the PPP after heme exposure

**Figure 4:**
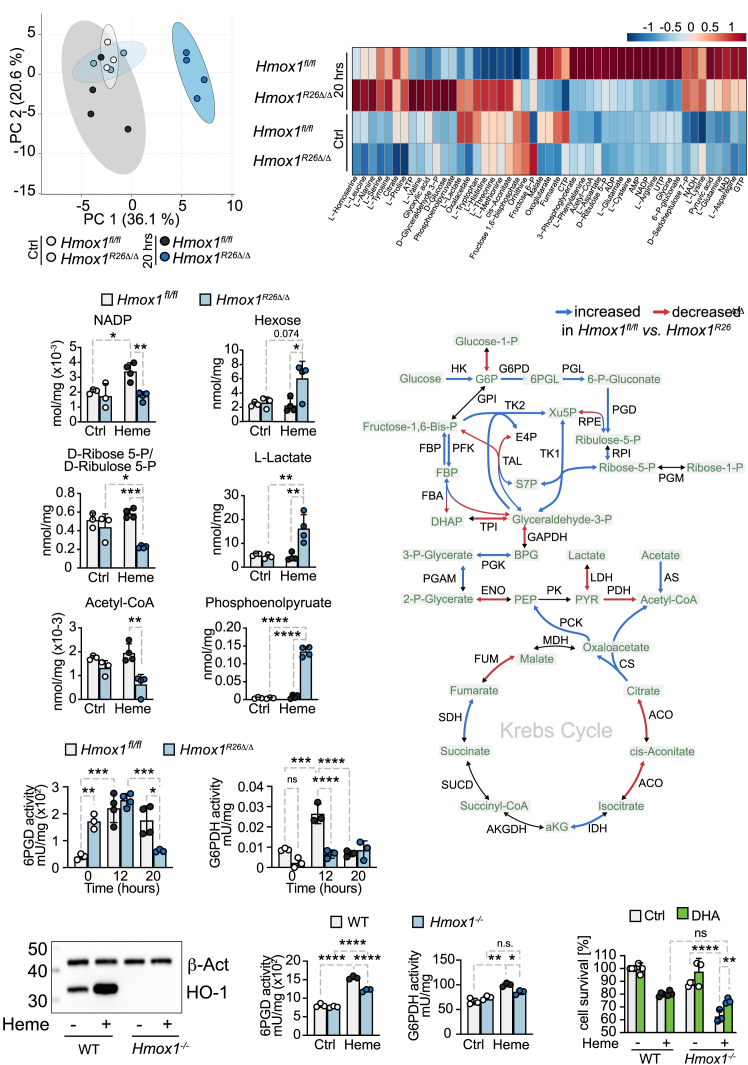
*Hmox1* deletion impairs pentose phosphate pathway in kidneys (A) Principal component analysis (PCA) for kidney metabolites. Data show difference between 14 samples of non-stimulated or 20 h after heme-stimulation (four different conditions with each with *n* = 3–4 animals per group). (B) Heatmap analysis highlighting metabolites after normalization of non-stimulated animals or 20 h after heme. *n* = 3–4 animals per group. (C) Targeted metabolomics of the kidney and (D) targeted flux balance analysis for ATP generation using data integration into a reconstructed metabolic network. (E) Enzymatic activity of 6PGD and G6PDH in total kidney lysates from *Hmox1*^*fl/fl*^ and *Hmox1*^*R26Δ/Δ*^ mice at baseline or 20 h after hemin injection. (F) Western blot, (G) enzymatic activity of 6PGD or enzymatic activity of 6GPDH from *Hmox1*^*+/+*^ and *Hmox1*^−/−^ TKPTS cells with and without heme stimulation. (H) Cell survival *Hmox1*^*+/+*^ and *Hmox1*^−/−^ TKPTS cells treated or not with dehydroascorbic acid (DHA). Data are shown as mean ± SD (*n* = 3–4 mice per group). Dots represent individual animals/experiments. ∗*p* < 0.05; ∗∗*p* < 0.01; ∗∗∗*p* < 0.001; ∗∗∗∗*p* < 0.0001. AC, acetate; ACCOA, acetyl-CoA; ACON, aconitate; Act, actin; AKG, ⍺-ketoglutarate; BPG 1, 3-bisphosphoglycerate; CIT, citrate; DHA, dehydroascorbic acid; DHAP, dihydroxyacetone phosphate; E4P, erythrose 4-phosphate; FDP, fructose 1; 6-bisphosphate; FUM, fumarate; F6P, fructose 6-phosphate; G1P, glucose 1-phosphate; G6P, glucose 6-phosphate; G3P, glyceraldehyde 3-phosphate; ICIT, isocitrate; LAC, lactate; MAL, malate; ns, non-significant; OAA, oxaloacetate; PC, principal component; PEP, phosphoenolpyruvate; PYR, pyruvate; Ru5P, ribulose 5-phosphate; R1P, ribose 1-phosphate; R5P,. ribose 5-phosphate; SUCC, succinate; SUCCOA, succinyl-CoA; S7P, sedoheptulose 7-phosphate; WT, wild-type; XuP5, xylulose 5-phosphate; 2PG, 2-phosphoglycerate; 3PG, 3-phosphoglycerate; 6PGC, 6-phosphogluconate; 6PGL, 6-phosphogluconolactone.

We then sought to investigate the role of *Hmox1* expression in supporting cellular metabolism adaptation to heme-induced stress. To accomplish this, we conducted targeted metabolomics analyses of energy metabolism-related compounds and amino acids in the kidneys and livers of both *Hmox1*^*R26Δ/Δ*^ and *Hmox1*^*fl/fl*^ mice, both before and 20 h after *i.p*. hemin administration (Figures 4A*–*4C and S7–S9). Metabolomic profiling of renal tissue demonstrated a modest enrichment (enrichment ratio <2) of metabolites associated with lipid metabolic pathways under steady-state conditions (Figure S7A). However, after hemin application, a more substantial enrichment (enrichment ratio 6) was observed for metabolites associated with glutamine, nitrogen, and porphyrin metabolisms (Figure S7B). Next, we integrated these data into the genome-scale metabolic reconstruction of mouse metabolism, iMM1865,^48^ and analyzed it using flux variability analysis.^49^ In the kidney, we observed activity in central carbon metabolism, including glycolysis, the PPP, and the tricarboxylic acid (TCA) cycle. We had previously shown that heme/iron metabolism impacted hepatic gluconeogenesis in animals with bacterial sepsis and malaria.^4^^,^^22^ In this experimental setup, glycolysis showed an inconsistent picture of induced as well as reduced fluxes in *Hmox1*^*R26Δ/Δ*^ vs. *Hmox1*^*fl/fl*^ for both kidney and liver (Figures 4D and S8D). We observed notable differences in the PPP between *Hmox1*^*R26Δ/Δ*^ and *Hmox1*^*fl/*fl^ animals, with the PPP showing decreased flux capabilities in kidney (Figures 4A–4D). Determination of the enzymatic activity of two key enzymes of the PPP, 6-phosphogluconate dehydrogenase (6PGD), and 6GPDH revealed that control animals but not *Hmox1*^*R26Δ/Δ*^ can mount an adaptive increase in their activity in response to hemin application (Figure 4E).

The hepatic PPP displayed a similar trend, except for transketolase and 6-phosphogluconolactonase, suggesting that *Hmox1* expression preserves PPP activity (Figures S8 and S9). Phenotyping data analyses (Figure 1) indicated an inability of *Hmox1*^*R26Δ/Δ*^ animals to meet their energy demands: Metabolomic analysis further elucidated that increased energy demands could be met in the liver but not in the kidney. This is supported by significantly elevated ATP levels in the livers of *Hmox1*^*R26Δ/Δ*^ mice, although this was associated with signs of liver failure. These findings suggest that the liver adapts effectively to heme-mediated stress, regardless of *Hmox1* expression (Figures 1, S8B, and S8C). Furthermore, fluxes through citrate synthase and phosphoenolpyruvate carboxykinase were increased in the kidneys but decreased in livers of *Hmox1*^*R26Δ/Δ*^ compared to *Hmox1*^*fl/fl*^ animals (Figure S8D). This points toward tissue-specific regulation of gluconeogenesis. To further confirm our observations, we performed a genetic knockout of *Hmox1* in murine Immortalized Mouse Kidney Proximal Tubule Epithelial Cells (TKPTS) cells, a kidney cell line (Figure 4F), and assessed the activity of the same two enzymes, 6PGD and 6GPDH. At baseline, no difference in enzymatic activity was observed between genotypes. Following hemin exposure, a decreased activity of enzymes regulating the PPP, namely 6PGD and 6GPDH was noted in *Hmox1*^−/−^ cells only, confirming the *in vivo* and *in silico* data (Figure 4G). Having shown, that *Hmox1* expression in renal proximal tubular epithelial cell (RPTEC) cells promotes PPP, we then rescued *Hmox1*^−/−^ cells from heme-induced stress with dehydroascorbate, which has been shown to stimulate the PPP pathway and NAPDH generation (Figure 4H).

## Discussion

Our study provides experimental evidence how *Hmox1* expression controls cellular metabolism. We can demonstrate that under physiological conditions, HO-1 is not required for maintaining organ function, likely owing to a compensatory activity of the HO-2 isoenzyme. However, under conditions associated with increased levels of labile heme, such as hemolysis,^9^ a rapid upregulation of *Hmxo1* becomes indispensable to prevent heme-induced organ dysfunction. Our findings confirm earlier data that kidneys are most sensitive to labile heme.^27^^,^^32^^,^^50^ Time-resolved analysis of RNA sequencing data from kidney reveals that *Hmox1* expression was associated with a distinct transcriptional response to heme. Specifically, the Wnt/β-catenin and the oxidative phosphorylation pathways were oppositely regulated in kidneys of *Hmox1*^*R26Δ/Δ*^ and control mice.

Transient activation of Wnt/β-catenin, as observed in control mice essentially promotes repair and recovery, while its sustained activation triggered maladaptive responses and was associated with increased kidney injury.^27^^,^^50^ It is not entirely clear how and if these two pathways interact during heme-induced stress. Potentially, heme-exposure induces a metabolic shift into oxidative phosphorylation to maintain cellular ATP content with the detriment of tissue repair-promoting Wnt/β-catenin pathway. While *Hmox1* expression in the kidney allows to balancing ATP needs, the adaptive capacity is insufficient in *Hmox1*^*R26Δ/Δ*^ mice. This could lead to a viscous circle with further increased ATP demands, accelerated acute kidney damage, systemic energetic crisis, and organismal death.

Using targeted metabolomics and *in silico* analysis to predict the metabolic flux for central carbon metabolism, we observed an increased PPP flux in *Hmox1*^*fl/fl*^ but not in *Hmox1*^*R26Δ/Δ*^ animals upon heme exposure. This results in a tissue-specific increase in NADP content in the kidney but not in liver. Interestingly, the reverse was observed for ATP content, which was not affected in kidneys but increased in the liver. This suggests that tissues have different capacities to maintain homeostasis and function when challenged by labile heme. This suggests a *Hmox1* involvement in directing glucose metabolism through the PPP to enhance NADPH production, supporting antioxidant defenses, and facilitating tissue repair processes.^51^^,^^52^^,^^53^ Conversely, the lack of such an adaptive increase in PPP flux in *Hmox1*-deficient mice underscores its essential role in maintaining cellular energetics and redox balance under oxidative stress. This adaptive increase is not observed in *Hmox1*^*R26Δ/Δ*^ animals. Notably, metabolomics-driven simulated fluxes in the TCA cycle differed only partially between genotypes indicating that the differing metabolome profile affected early steps in one-carbon metabolism. This set of data also suggests that *Hmox1* expression facilitates glucose shuttling via the PPP to optimize the metabolic stress response. Likely, increased PPP flux is a direct way to enhance NADPH production from glucose. This would improve the host response, e.g., via supporting antioxidant enzymes that use NADP as a prosthetic group^54^ and via promoting reductive biosynthesis facilitating tissue repair.^51^^,^^52^^,^^53^ Less likely is an adaptation to increased NADP contents derived from *Hmox1*-mediated heme degradation.^54^ In macrophages, enhanced flux through the PPP has been shown to support the inflammatory response by promoting superoxide production.^53^^,^^55^^,^^56^^,^^57^ However, how this mechanism translates to the renal response remains unclear. We found a significant upregulation of genes involved in the PPP in response to heme independently from the expression of *Hmox1* but higher fluxes in *Hmox1*-expressing animals. Since heme induces lipid-peroxidation, and NADPH might be required for lipid synthesis, the potential to increase NADPH production would allow producing cellular lipids and replenishing the lipid peroxidation pool. Potentially, reaction intermediate metabolites are used to counter ROS production, and lipid peroxidation. Consistent with this, our RNA sequencing data reveal upregulation of pathways that mitigate oxidative stress by promoting metabolic energy production, or correcting protein translation.^58^^,^^59^^,^^60^^,^^61^^,^^62^

An even more complex picture of the cellular adaptive response arises when considering specific metabolites. Targeted metabolomics data show a decrease in cellular glycine in response to heme in *Hmox1*^*R26Δ/Δ*^ and an increase in control animals. Concomitantly, there was no difference in serine concentrations between genotypes before or after hemin application. This suggests that serine/folate-mediated one-carbon metabolism is not utilized as a carbon donor in our experimental settings.^63^ Instead, as proposed before, glycine will be oxidized to produce additional NADPH^64^ to compensate for increased needs. In light with decreased acetyl-CoA contents and considering increased transcription of genes countering lipid-peroxidation and promoting lipid biosynthesis,^65^ this supports the notion of increased needs for lipogenesis due to enhanced heme-induced lipid peroxidation and dysfunctional heme degradation. Heme exposure appears to significantly affect glutathione and L-arginine metabolism, both promoting redox homeostasis.^63^^,^^66^ Finally, our findings implicate a place for HO-1 in the regulatory network that modulates G6PD activity, a critical enzyme in the PPP, thereby influencing the pathway overall flux. This regulatory effect might be mediated through the activation of Nrf2, a master regulator of cellular antioxidant response. Nrf2 activation by products of HO-1, such as carbon monoxide and biliverdin, can shift glucose metabolism toward the PPP, enhancing NADPH production essential for countering oxidative stress.^67^^,^^68^

Our data might also have further implications. Both, *HMOX1* expression^69^^,^^70^^,^^71^ and the PPP were increased in models of ischemia reperfusion injury.^72^ Also, the expression of G6PD, the initial and rate-limiting PPP enzyme, and induction of the PPP promoted neuroprotection in brain ischemia.^73^ The protective properties have mostly been assigned to the generation of carbon monoxide^74^ or biliverdin.^75^ Our results suggest that additionally, removal of heme by HO-1 is an important mechanism to maintain necessary PPP function in ischemia reperfusion injury. This might involve activation of the master regulator of the antioxidative stress response, Nrf2 by heme, whose activation can shift glucose metabolism into PPP resulting in increased NADPH content.^67^^,^^68^^,^^76^ Furthermore, the results obtained from the knockout of *Hmox1* in RPTEC cells, further support the role of *Hmox1* in maintaining PPP activity. The reduction in PPP enzymatic activity in *Hmox1*-deficient cells suggests that the absence of *Hmox1* leads to a functional impairment of the PPP. The absence of HO-1-mediated heme degradation could exacerbate lipid peroxidation, potentially disrupting Nrf2 signaling and its downstream protective effects. Experimental data from HO-1-deficient TKPTS cells support this hypothesis, revealing a decline in G6PD enzymatic activity. Such a decline suggests that expression of *HMOX1* maintains PPP efficiency, highlighting its significance in the broader context of cellular antioxidative mechanisms and metabolic homeostasis.

### Limitations of the study

Our findings can advance the understanding the role of *Hmox1* role in regulating heme-induced stress. However, several limitations need to be acknowledged.

(1) Our data do not permit a definitive assessment of *Hmox1* function at the level of individual cell types, as not all immune and non-immune cell populations, e.g., in the kidney were evaluated. Our findings however suggest that neither myeloid cells nor proximal tubular epithelial cells alone account for the full HMOX1-related effects that we report. The observation that *Hmox1* deficiency specifically in these cell types does not result in enhanced mortality after hemin application may reflect an intercellular metabolic compensation,^77^ in which neighboring cells are able to share metabolites or buffer their deficiencies. Another possibility is that residual *Hmox1* activity in non-deleted tissues may be sufficient to prevent the phenotype that is seen after ubiquitous deletion.
(2) Another limitation is the lack of experimental evidence demonstrating that the observed phenotype results from direct heme-induced toxicity and not from reduced availability of HO-1 reaction byproducts other than iron, specifically biliverdin and carbon monoxide.
(3) Metabolic assessments were conducted in two principal organs, liver and kidney. Nevertheless, it remains plausible that *Hmxo1* deficiency could impair function of additional metabolically demanding tissues-notably the myocardium, which exhibits a particularly high dependence on oxidative ATP generation and O_2_ consumption.
(4) We primarily utilize a conditional *Hmox1* knockout mouse model. Although this approach yields valuable insights into the functions of HO-1, the findings may not fully reflect the complexity of *Hmox1* activity in humans or other species. Finally, while our study elucidates the outcomes of *Hmox1* deletion on metabolic and inflammatory pathways, the precise molecular mechanisms underlying these effects remain only partially defined. A deeper exploration of the signaling pathways and cellular interactions mediated by *Hmox1* might be crucial for a complete understanding of its protective mechanisms.

In conclusion, our data delineate a crucial role for HO-1 in orchestrating the renal metabolic response to labile heme. By controlling PPP activity, HO-1 facilitates a protective metabolic adaptation, essential for kidney function under hemolytic stress. This study highlights the significance of HO-1 in modulating PPP flux and enriches our understanding of the pathophysiological processes underlying kidney dysfunction in hemolytic conditions.

## Resource availability

### Lead contact

Further information and requests for reagents and resources can be directed to, and will be fulfilled by, the lead contact, Sebastian Weis (sebastian.weis@med.uni-jena.de).

### Materials availability

All unique and stable reagents generated in this study are available from the lead contact with a completed materials transfer agreement.

### Data and code availability

• The raw data files for the RNA sequencing and analysis have been deposited as GEO: GSE246570; NCBI tracking system #24354011.
• This paper does not report original code.
• Any additional information required to reanalyze the data reported in this paper is available from the lead contact upon request.

## Acknowledgments

J.G., E.J., V.H., M.B., and S.W. were supported by the Integrated Research and Treatment Center—10.13039/501100013457Center for Sepsis Control and Care (CSCC) at the 10.13039/501100007653Jena University Hospital. The CSCC was funded by the German Ministry of Education and Research (10.13039/501100002347BMBF no. 01EO1502). S.S. and G.P. were supported by the 10.13039/501100001659DFG CRC/Transregio 124 “Pathogenic fungi and their human host: Networks of interaction” (10.13039/501100001659DFG project number 210879364, subproject INF). S.W. was funded by the 10.13039/501100001659Deutsche Forschungsgemeinschaft (DFG, German Research Foundation) DFG, project number WE 4971/6-1 and WE 4971/9-1. M.P.S. is funded by the Gulbenkian foundation and the following research grants, “10.13039/100010434la Caixa” (HR18-00502) and 10.13039/501100001871FCT (5723/2014; FEDER/29411/2017; PTDC/MED-FSL/4681/2020; 2022.02426.PTDC), Oeiras-ERC Frontier Research Incentive Awards, SymbNET Research Grants (H2020-WIDESPREAD-2020-5-952537), and Congento (LISBOA-01-0145-FEDER-022170). E.J. was funded by the 10.13039/501100001659Deutsche Forschungsgemeinschaft (DFG, German Research Foundation) under Germany's Excellence Strategy—EXC 2051—project-ID 390713860 and by the 10.13039/501100001659Deutsche Forschungsgemeinschaft, DFG as well as by 10.13039/501100001659Deutsche Forschungsgemeinschaft (DFG) grant GRK 1715/2. E.J. is currently funded by “10.13039/100010434la Caixa” (HR18-00502). T.W. was supported by 10.13039/501100001659Deutsche Forschungsgemeinschaft (DFG, SFB1192).

## Author contributions

J.G. performed experiments and analyzed the data; W.V. performed FACS analysis; D.M-.A. performed *in vitro* experiments, cell culture experiments, western blots, and enzymatic assays; E.J. performed and analyzed ELISAs, assessed cytokine release, and performed and analyzed mouse experiments; F.R. performed and analyzed cell culture and flow cytometry experiments; G.H. performed quantitative reverse-transcription PCR (RT-qPCR) analysis; S.S., G.P., B.P.S., and M.G. performed and analyzed metabolomics-driven genome-scale metabolic modeling simulations and performed RNA sequencing and data analysis. V.H. and J.G. performed magnetic resonance imaging (MRI). E.K. did and analyzed multiplex ELISAs. T.W. conducted and evaluated histological analyses. M.P.S. contributed to study hypothesis and design and provided mouse strains. M.B. provided critical input and participated in writing of the manuscript. S.W. and J.G. formulated the initial hypothesis, designed the experiment, analyzed data, and wrote the manuscript. All authors edited, read, and approved the manuscript.

## Declaration of interests

The authors declare no competing interests.

## STAR★Methods

### Key resources table

**Table:** 

REAGENT or RESOURCE	SOURCE	IDENTIFIER
**Antibodies**		

Goat anti rabbit IgG horseradish peroxidase (polyclonal)	Cell Signaling Technology, Massachusetts, USA	Cat#: 7074; RRID: AB_2099233
β-Actin antibody (clone 13E5) Rabbit mAb	Cell Signaling Technology, Massachusetts, USA	Cat#: 4970; RRID: AB_2223172
Heme Oxygenase 1 antibody (clone EPR1390Y)	Abcam, Berlin, Germany	Cat#: ab68477; RRID: AB_1267209
Anti-mouse Ly6G/Ly6C (Gr-1) (clone RB6-8C5)	BioXcell, New Hampshire, USA	Cat#: BE0075; RRID: AB_10312146
Rat IgG2b Isotype control (clone LTF-2)	BioXcell, New Hampshire, USA	Cat#: BE0090; RRID: AB_1107780
VioGreen anti-mouse CD45 (clone REA737)	Miltenyi Biotec, Cologne, Germany	Cat#: 130-110-803; RRID: AB_2658222
VioBlue anti-mouse CD11b (clone REA592)	Miltenyi Biotec, Cologne, Germany	Cat#: 130-113-810; RRID: AB_2726327
FITC anti-mouse Ly-6G (clone REA526)	Miltenyi Biotec, Cologne, Germany	Cat#: 130-120-820; RRID: AB_2784431
PE anti-mouse NK1.1 (clone REA1162)	Miltenyi Biotec, Cologne, Germany	Cat#: 130-120-506; RRID: AB_2752122
PEVio770 anti-mouse Ly-6C (clone REA796)	Miltenyi Biotec, Cologne, Germany	Cat#: 130-111-506; RRID: AB_2652810
APC anti-mouse F4/80 (clone REA126)	Miltenyi Biotec, Cologne, Germany	Cat#: 130-116-525 RRID: AB_2733417
APCVio770 anti-mouse CD3 (clone REA641)	Miltenyi Biotec, Cologne, Germany	Cat#: 130-119-793 RRID: AB_2751847

**Chemicals, peptides, and recombinant proteins**		

Deoxycholic Acid	Sigma Aldrich, St. Louis, USA	Cat#: D6750
Dimethylsulfoxide	ChemCruz, Dallas, USA	Cat#: sc.202581
EGTA	Sigma Aldrich, St. Louis, USA	Cat#: E4378
Fetal calf serum	ThermoFisher Scientific, Waltham, USA	Cat#: 10270106
Hemin	Frontier Scientific, Logan, USA	Cat#: H651-9
Kollisolv® PEG E 400	Sigma Aldrich, St. Louis, USA	Cat#: 06855
L-Glutamine, BioUltra, ≥99.5% (NT)	Sigma-Aldrich, St. Louis, USA	Cat#: 49419-25G
Laemmli Buffer 10x for SDS Page	Serva, Heidelberg, Germany	Cat#: 42556.02
Light Eos CL HRP WB Substrate Kit	Serva, Heidelberg, Germany	Cat#: 42585.02
Phenylhydrazine	Sigma Aldrich, St. Louis, USA	Cat#: P26252
Protease inhibitor cocktail	Sigma-Aldrich, St. Louis, USA	Cat#: P8340
Sivelestat (sodium tetrahydrate)	MedChem Express, New Jersey, USA	Cat#: HY-17443B

**Critical commercial assays**		

BCA Protein Assay Macro Kit	Serva, Heidelberg, Germany	Cat#: 11945.04
CountBrightTM Absolute Counting Beads	Invitrogen, Waltham, USA	Cat#: C36995
LegendPlex™ Mouse Inflammation Panel (13-plex)	BioLegend, San Diego, USA	Cat#: 740150
Glucose-6-phosphate Dehydrogenase Assay kit	Abcam, Cambridge, UK	Ca#: ab102529,
6-Phosphogluconate Dehydrogenase Assay Kit	Abcam, Cambridge, UK	Ca#: ab241016

**Experimental models: Organisms/strains**		

Mouse: *C57Bl/6J*	bred in house	originally from Jackson
Mouse: *C57BL/6J Hmox1*^*fl/fl*^	bred in house	originally from IGC, Portugal
Mouse: *C57BL/6 R26Cre-ERT2 Hmox1fl/fl*	bred in house	originally from IGC, Portugal
Mouse: *PepckCreHmox1*^*fl/fl*^	Bred and housed, IGC, Portugal	originally from (*27*)

**Oligonucleotides**		

Mouse Primer: *Arbp0* Fwd: 50CTTTGGGCATCACCACGAA30 Rev: 50GCTGGCTCCCACCT TGTCT3	Weis et al., 2017^4^	N/A
Mouse Primer: *Hmox1* Fwd: 5′TGACACCTGAGGTCAAGCAC3′ Rev: 5′TCTCTGCAGGGGCAGTATCT3′	Ramos et al.*,* 2019^28^	N/A

**Software and algorithms**		

MetaboAnalyst 5.0	MetaboAnalyst 5.0	MetaboAnalyst 5.0
Prism (GraphPad) Version 9		https://www.graphpad.com/
FlowJo 10.7.1		https://www.flowjo.com/
Gurobi Optimization, LLC 2021		https://www.gurobi.com/
ImageJ		https://imagej.nih.gov/ij/
Slicer 4.11.0		https://www.slicer.org/
GSEA 4.3.2		https://www.gsea-msigdb.org/gsea/downloads.jsp
R 4.0.4	Team R C. R: A language and environment for statistical computing[J]. 2013.	https://cran.r-project.org/bin/windows/base
RStudio (v1.1.463)	Team R S. Rstudio: integrated development for R[J]. Rstudio, Inc., Boston, MA URL http://www.Rstudio.com, 2015, 42: 14.	https://www.rstudio.com/products/rstudio/download/

**Deposited data**		

Raw and analyzed data, Transcriptomes	Raw and analyzed data, Transcriptomes	GEO: (GSE246570) [NCBI tracking system #24354011]

**Other**		

Rodent Thermometer	Bioseb	Cat#: BIO-TK8851
ACCU-CHEK Aviva 50ct Strip	Roche	Cat#: 06453970

### Experimental model and study participant details

#### Animal experiments

Experimental procedures were ethically reviewed and approved by the Ethics Committee of the regional animal welfare committee (Registration number: 02-048/16, Thuringian State Office for Consumer Protection and Food Safety) or the Instituto Gulbenkian de Ciência (license reference: A009/2011, by Direção Geral de Alimentação e Veterinária (license reference: 0420/000/000/2012). All experiments conducted on animals followed the German legislation on protection of animals, the Portuguese (Decreto-Lei n° 113/2013) and the European (Directive 2010/63/EU) legislations, concerning housing, husbandry and animal welfare. Mice were kept in two different animal facilities, both specific pathogen free (SPF). Animals were housed under controlled temperature (22-24°C) and humidity (40-50%) conditions on a 14 hours (hrs) light/10 hrs dark cycle. Mice were single-housed only when required by the experiment, *i.e.* fasting-refeeding, and indirect calorimetry. Mice were fed *ad libitum* with a standard rodent chow diet, containing 57% carbohydrates, 34% protein and 9% fat (Ssniff® Spezialdiäten GmbH, V1554) and had *ad libitum* access to water. Access to food was not allowed only for specific experiments.

#### Mouse models and induced *Hmox1* deletion

*ROSA26Cre-ERT2 Hmox1*^*flox/flox*^ “knock-in” mice (*Hmox1*^*R26fl/fl*^) in which the *Hmox1* allele is flanked by two LoxP sites, allowing for its ubiquitous deletion by Cre recombinase expression after Tamoxifen administration were used.^4^ Conditional deletion of the *Hmox1* allele in *Hmox1*^*R26fl/fl*^ mice was initiated in approximately six- weeks-old mice. *Hmox1*^*R26fl/fl*^ and control *Hmox1*^*flox/flox*^ mice were fed *ad libitum* Rat/Mouse diet with Tamoxifen (M-Z, Low phytoestrogen; 400 mg/kg TAM Citrate + Sucrose flavor, 10 mm Soybean free diet, sterilized 25 kGy; Ssniff Spezialdiäten GmbH) for 8 weeks. Successful *Hmox1* deletion was assessed by qRT-PCR of leukocytes collected from facial vein blood. Animals with less than approximately 10% remaining basal *Hmox1* mRNA content were used for further experiments (Figure S1A). Male and female animals were used.

*Hmox1*^*PepckΔ/Δ*^ were bred and deleted at the IGC, Oeiras, Portugal. Conditional deletion of the *Hmox1* allele in renal proximal tubular epithelial cell (RPTEC) was induced at 7 weeks after birth by the addition of 0.3M NH_4_Cl to drinking water for one week. *Hmox1*^*PepckΔ/Δ*^ mice were used earliest four weeks after exposure to acidified water.^22^

#### Cell culture and generation of *Hmox1* CRISPR–Cas9 knockout cell line

TKPTS (a cell line from mouse proximal tubule of the kidney) were cultured in DMEM medium supplemented with 10 % (v/v) fetal bovine serum (FBS) and 1% (v/v) Penicillin-Streptomycin at 37°C, 95% humidity, 5 % CO2. Stimulation with hemin was performed in DMEM medium supplemented with 10% (v/v) FBS and 1 % (v/v) Penicillin-Streptomycin. TKPTS cells were transfected with 2 μg CRISPR–Cas9 expressing knockout plasmids (sc-420882 from Santa Cruz) using Lipofectamin^TM^ 2000 transfection reagent (ThermoFisher) as described by the manufacturer. The plasmids used for gene knock-out were a mixture of three plasmids, each carrying a different guide RNA specific for the target gene, as well as the Cas and GFP coding regions. GFP+ cells were selected by sorting on a BD FACSAria^TM^ Fusion (BD Biosciences) 48 hrs after transfection. Deletion of target protein expression was verified by Western blot.A All cell cultures were routinely checked for Mycoplasma contamination.

### Method details

#### Hemin preparation

Stock solution of Heme (Frontier Scientific) was prepared as described in.^78^ Briefly, hemin was dissolved in 0.2 M NaOH and adjusted to a pH of 7.4 followed by centrifugation at 3,000 *g*; 15 min 4 °C and filtration with a 70 μm cell strainer. Concentration of stock solution was determined by measuring OD 405 nm of a 1:1,000 dilution in DMSO. Concentration was calculated using the Lambert-Beer law.

#### *In vivo* experiments

##### Hemin, N-Acetylcysteine and sivelestat applications

Hemin (Frontier Scientific) stock solutions were diluted in 150 μL PBS. 20 mg/kg body weight were injected *i.p.* into mice. N-Acetylcysteine (NAC) (Fulka) stock solution was prepared in PBS with its p.H. adjusted to 7.2. This solution was administered *i.p.* at a concentration of 100 mg/kg body weight every 12 hrs, one day prior to hemin administration and every 12 hrs henceforth. Sivelestat (MedChemExpress) stock solution was prepared in a solution of 10% DMSO (Chemcruz), 40% PEG 400 (Sigma), 5 % Tween 80 (Sigma) and 45% PBS. This solution was administered *i.p.* at a concentration of 100 mg/kg body weight six hrs after hemin/phenylhydrazine (PHZ) administration and once daily henceforth. Ferrostatin-1 (MedChemExpress) stock solution was prepared in a solution of 10% DMSO (Chemcruz), 40% PEG 400 (Sigma), 5% Tween-80 (Sigma) and 45% PBS. This solution was administered *i.p.* at a concentration of 10 mg/kg body weight, every 12 hrs one day before heme/PHZ administration and every 12 hrs henceforth. Iron-dextran was diluted in 200 μL PBS and injected i.p. at a concentration of 1.8 mg/kg corresponding to iron in 20 mg/kg hemin.

##### Intravascular hemolysis

Intravascular hemolysis was induced by a single *i.p.* injection of PHZ with 50 mg/kg body weight. Survival was assessed twice daily for 3 days and once daily for the next 9 to 12 days. Body weight, rectal temperature, and blood glucose levels were monitored daily for up to 7 days.

##### Granulocyte depletion

Mice were injected *i.p.* with 10 mg/kg of anti-GR-1 (clone RB6-8C5, Bioxcell), or isotype control (rat IgG2b, clone LTF-2, Bioxcell) in 150 μL PBS 24 hrs prior to and 3, 6, 9 and 12 days after stimulation (day 0). Depletion efficiency was determined using single-cell suspensions of spleen, bone marrow, and blood. The percentage of GR-1 positive cells was determined by flow cytometry using the following antibodies: CD45/VioGreen (clone REA737), CD3/APC-Vio 770 (clone REA641), NK1.1/PE (clone REA1162), CD11b/VioBlue (clone REA592), Ly-6G/FITC (clone REA526), F4/80/APC (clone REA126), and Ly-6C/PE-Vio 770 (clone REA796) (Miltenyi Biotec). Cells were stained with propidium iodide (Sigma-Aldrich) for 5 min at room temperature (RT) after cell surface staining to exclude dead cells in the analysis. For absolute quantification, CountBright^TM^ Absolute Counting Beads (Invitrogen) kit was used according to the manufacturer’s instructions. Isotype antibodies and fluorescence minus one (FMO) control were used for gating purposes. Fluorescent intensity was determined using a FACSAriaTM Fusion cell sorter (BD Biosciences) and the data were analyzed using FlowJo 10.7.1 software (TreeStar).

##### Tolerance tests

Before performing a tolerance test, mice were deprived of food for 16 hrs overnight, plus cage change at time of fasting. D-glucose (2 mg per g body weight in 200 μL drinking water) was administered by oral gavage. Alanine and glutamine (2 mg per g body weight in 200 mL sterile 0.9% saline), were injected *i.p.*, as well as insulin (0.75 IU insulin per kg body weight). Blood glucose from the tail vein was measured with a glucometer before and after gavage/injection at the indicated time points.

#### Metabolic cages

Promethion Core (Sable Systems) was used to measure indirect calorimetry. After two days of acclimation phase, experimental procedures were performed. The recording continued for the following seven days. The system consists of a standard GM-500 cage with a food hopper and a water bottle connected to load cells (2 mg precision) with 1 Hz rate data collection. Additionally, the cage contains a red house enrichment. Total (ambulatory and fine) activity was monitored at 1 Hz rate using an XY beam break array (1 cm spacing). Oxygen, carbon dioxide and water vapor were measured using a CGF unit (Sable Systems). This multiplexed system operated in pull-mode. Air flow was measured and controlled by the CGF (Sable Systems) with a set flow rate of 2 L/min. Oxygen consumption and carbon dioxide production were reported in milliliters per minute (mL/min). Energy expenditure was calculated using the Weir equation.^79^ Respiratory Exchange Ratio (RER) was calculated as the ratio of VCO_2_/VO_2_. Raw data was processed using Macro Interpreter v2.38 (Sable Systems).

#### Cytokine measurements

Plasma cytokines were determined using the LEGENDplex™ Mouse Inflammation Panel 13-plex (BioLegend, San Diego), according to manufacturer instructions.

#### Magnetic Resonance Imaging

Magnetic Resonance Imaging (MRI) was used to detect signal cancellation induced by iron and to quantify the corresponding T2 relaxation time. Briefly, longitudinal MRI was performed every 3 months, after tamoxifen diet. Images were acquired at 9.4 T on a Bruker BioSpec 94/20 system equipped with a 0.7 T/m gradient system and ParaVision 6.0.1 operating software (Bruker BioSpin). A 72-mm volume resonator coil was used for signal transmission. A four channel receive coil array was used for signal reception. During MRI, the animals were anesthetized with isoflurane (1.5–2.5% isoflurane and 0.7/0.3 air/O2 mixture), and core body temperature and respiration rates were monitored using an MRI compatible monitoring system (SA Instruments, Stony Brook, NY). Respiratory-gated anatomical MRI of the abdomen was performed in axial view using a self-gated cine FLASH sequence (IntraGate, Bruker BioSpin MRI) with the following parameters: TR: 8 ms, TE: 2.9 ms, FA: 10<°, number of repetitions 100, FOV: 30 x 30 mm2, Matrix: 256 x 256, slice thickness: 1 mm.

#### Serology

Mice were euthanized using CO_2_ inhalation, and blood was collected by cardiac puncture using a heparinized syringe. Blood samples were stored for 30 min at 4°C and subsequently centrifuged at 2000 g, 10 min, 4°C. The plasma was then collected into a fresh 1.5 mL Eppendorf tube and stored at -80°C until further analysis. Measurements of conventional serological parameters were performed by SYNLAB.vet GmbH (Leipzig).

#### Western blot

Homogenized tissue samples were washed twice with ice cold 1x PBS before the addition of ice cold RIPA buffer (Tris-HCl (50 mM) p.H 7.5, NaCl (150 mM), EGTA (1 mM), IGEPAL (1% v/v), Deoxycholic acid (0.25% w/v), Na_3_VO_4_ (1 mM), NaF (1 mM), protease inhibitor cocktail (Sigma) and phosphatase Inhibitor Cocktail 3 (Sigma). Lysates were maintained on ice before being centrifuged at 16,000 *g* for 10 min at 4 °C and the supernatant collected for subsequent determination of protein concentration via the BCA method (Serva). An amount of 15 μg of total protein was mixed in 1x Laemmli buffer and denatured at 95 °C for 5 min. Proteins were resolved by SDS-polyacrylamide gel electrophoresis, transferred to 0.2 mm PVDF membranes, by semi-dry transfer. PVDF membranes were blocked for 30 min in Tris-buffered Saline containing Tween 20 (TBST) with 5% (w/v) BSA, at room temperature. All primary antibodies were used at a concentration of 1:1000 and incubated overnight at 4°C. After overnight incubation, membranes were washed for 30 min at room temperature in TBST, incubated with the appropriate secondary antibody for 2 hrs at room temperature, and washed for a further 30 min in TBST at room temperature before developing. Proteins were detected by chemiluminescence in an ImageQuant LAS 4000 series machine and specific bands quantified using the open source FIJI software. Primary antibodies used were Heme Oxygenase 1 antibody (clone (E9H3A), Cell Signaling; AB_2893444), Heme Oxygenase 1 antibody (clone (EPR1390Y), Abcam; AB_1267209) and β-Actin antibody (Cell Signaling; AB_2223172). Secondary antibody used was goat anti rabbit IgG horseradish peroxidase (Cell Signaling; AB_2099233)).

#### qRT-PCR

RNA was isolated from tissues using mirVanaTM miRNA Isolation kit (ThermoFischer Scientific), according to the manufacturer’s recommendations. cDNA synthesis was accomplished using NZY M-MuLV First-Strand cDNA synthesis kit (NZYTech) from 1 ng of total RNA. Reverse transcriptase quantitative PCR (RT-qPCR) was performed using NZY qPCR green master mix (NZYTech) in a Rotor-Gene Q (QIAGEN) under the following conditions: 95°C/10 min, 40 cycles/95°C/15 s, annealing at 60°C/30 s and elongation 72°C/30 s. The following primers were used: *Hmox1* Fwd: 5′TGACACCTGAGGTCAAGCAC3’; Rev: 5′TCTCTGCAGGGGCAGTATCT3’^22^; *Arbp0*: Fwd: 5′CTTTGGGCATCACCACGAA3’; Rev: 5′GCTGGCTCCCACCTTGTCT3’^4^ *Ldhb* Fwd: 5′CCATGCTGAAAAACCTCTCCCG3’; Rev: 5′AGCGACCTCATCGTCCTTCAG3’; *Mdh1* Fwd: 5′TTCTGGACGGTGTCCTGATGGA3’; Rev: 5′TAGGACAGCCACATCCAGGTCT3’ (Origene); *Ccl2* Fwd: 5′CTGTTCACAGTTGCCGGCTG3’; Rev: 5′AGCTTCTTTGGGACACCTGCT3’; *Cox6b*1 Fwd: 5′GAACTGTTGGCAGAACTACCTGG3’; Rev: 5′ATGACACGGGACAGAGGGACTT3’ (Origene); *Uqcr11* Fwd: 5′TTTCTAGGCCCGCGCTACC3’; Rev: 5′GCACCCAGTCCAGGATCAGC3’; *Kat2a* Fwd: 5′CACGGAAATCGTCTTCTGTGCC3’; Rev: 5′CGTACTCGTCAGCATAGGTGAG3’ (Origene); *Trp53* Fwd: 5′CAAGATCCGCGGGCGTAAAC3’; Rev: 5′ATGGCGGGAAGTAGACTGGC3’; *Ccnd2* Fwd: 5′GCAGAAGGACATCCAACCGTAC3’; Rev: 5′ACTCCAGCCAAGAAACGGTCCA3’ (Origene).

#### RNA sequencing

Total RNA was extracted using *mir*Vana miRNA Isolation Kit (ThermoFisher Scientific). Sequencing of RNA samples was performed using Illumina’s next-generation sequencing methodology.^80^ Total RNA was quantified, and quality checked using Agilent 2100 Bioanalyzer Instrument (Agilent RNA 6000 Pico). Libraries were prepared from 500 ng of input material (total RNA) using NEBNext Ultra II Directional RNA Library Preparation Kit in combination with NEBNext Poly(A) mRNA Magnetic Isolation Module and NEBNext Multiplex Oligos for Illumina (96 Unique Dual Index Primer Pairs) following the manufacturer’s instructions (New England Biolabs). Quantification and quality checked of libraries was done using an Agilent 4200 TapeStation Instrument and D1000 ScreenTapes (Agilent Technologies). Libraries were pooled and sequenced in two NovaSeq6000 SP 100 cycle runs. System run in 101 cycle/single-end/standard loading workflow mode. Sequence information was converted to FASTQ format using bcl2fastq v2.20.0.422. Sequence read quality was assessed by means of the FastQC method (v0.11.5; http://www.bioinformatics.babraham.ac.uk/projects/fastqc/). Trimmomatic version 0.36 was used to trim Illumina adapters and poor-quality bases (trimmomatic parameters: leading=3, trailing=3, sliding window=4:15, minimum length=40).^81^The remaining high-quality reads were used to align against the Genome Reference Consortium mouse genome build 38 (GRCm38).^82^ Mapping was performed by HISAT2 version 2.1.0 with parameters as default.^83^

Count data were generated by means of the HTSeq method and analyzed using the DESeq2 method in the R statistical computing environment.^84^^,^^85^^,^^86^ Statistically significant differences were defined by Benjamini & Hochberg adjusted probabilities <0.05. Functional implications of the differentially expressed genes, were analysed by conducting gene enrichment analysis using the ‘enrichGO' function from the clusterProfiler package in R. The genes considered for enrichment analysis were identified as differentially expressed genes with a log2 fold <1. The enrichment analysis focused on identifying significantly enriched Gene Ontology (GO) terms associated with the differentially expressed genes in terms of biological processes (ont = ‘BP'). A p-value cutoff of 0.05 and a q-value cutoff of 0.05 were employed to determine the statistical significance of enriched terms (Table S1).

Functional enrichment analysis was conducted using the GSEA v4.3.2 along with MSigDB v2023.1.Mm. Gene set enrichment analysis (GSEA) based on the rankings of logFC of all genes and over-representation analysis was utilized to determine enriched biological process (BP) GO terms and use of the MSigDB Hallmark gene set. A cut-off of the adjusted p-value by the BH method was set to 0.05.

#### Protein-protein interaction network construction

The protein-protein interaction network to unveil both specific and nonspecific interactions among proteins, was constructed based on the differentially expressed genes with a combined score exceeding 0.4 in STRING. This threshold ensured that the interactions considered were functionally relevant. The network was assembled using the Cytoscape software (version 3.8) coupled with stringAPP (version 2.0.1).

#### Flow cytometry analysis

Single-cell suspensions from the spleen and bone marrow were obtained for cell surface staining. For bone marrow isolation, the femur was removed from mice after euthanasia, and ligament and muscles were mechanically cleared. The femur was separated in two pieces and bone marrow was collected by centrifugation in 1.5 mL microcentrifuge tubes. Spleen were harvested from mice, cut into small pieces and passed through a 70-μm cell strainer. Single-cell suspensions were stained in 5 mL polystyrene tubes in FACS buffer (2% BSA + 2mM EDTA + 2mM NaN3) with the following anti-mouse antibodies to identify distinct immune cell populations: CD45 (clone REA737), CD3 (clone REA641), NK1.1 (clone REA1162), CD11b (clone REA592), Ly-6G (clone REA526), F4/80 (clone REA126), and Ly-6C (clone REA796) (Miltenyi Biotec) for 20 minutes at 4 °C. Cells were stained with propidium iodide (Sigma-Aldrich) for 5 mins at RT after cell surface staining to exclude dead cells in the analysis. For absolute quantification, the CountBright™ Absolute Counting Beads (Invitrogen) kit was used according to the manufacturer's instructions. Fluorescence minus one (FMO) control was used to identify positive populations in the analysis. Samples were acquired on a FACSAria™ Fusion cell sorter (BD Biosciences) and analyzed with FlowJo 10.7.1 software (TreeStar).

#### Histology

Mice were perfused with 10 mL ice cold PBS. Liver, spleen and kidney samples were harvested, fixed in 4% buffered formaldehyde for 48 hrs at room temperature, then embedded in paraffin. Paraffin-embedded organ sections (4 μm) were dried on slides, deparaffinized in Xylol and rehydrated in a descending series. Tissue sections were then stained with hematoxylinand eosin, Prussian blue or PAS picrosirius red. Whole sections were analyzed, and images acquired using brightfield microscopy with a ZEISS Axio Imager 2. Tubular dilatation, dilatation of the peritubular capillaries (PTC), epithelial flattening, brush border loss, vacuolization and inflammatory cell presence was graded by an independent nephropathologist (T.W.) who was unfamiliar with the data in a pseudo-blinded manner.

#### Targeted metabolomics

Targeted metabolomics for amino acid and glucose metabolism metabolites was performed by Metabolomic Discoveries © (Potsdam, Germany) using high-throughput gas chromatography–mass spectrometry and accurate mass Quadrupole Time-of-Flight coupled to an Ultra Performance Liquid Chromatography.

#### Metabolomic pathway analysis

Pathways analysis on the datasets from the obtained metabolites was performed independently for every dataset using Mummichog 38 from the MetaboAnalyst 5.0 workflow. The pathway analysis was completed based on the default quantitative enrichment analysis method and the *Mus musculus* KEGG database. *In silico* analysis of differentially activated reactions in carbon metabolism relevant pathways was accomplished using the genome-scale mouse metabolic model iMM1865 28. All simulations were done using COBRApy (v0.20.0) 39 (with python3 (v3.6.12) and gurobi optimization (v9.1.1). First, an initial flux distribution was calculated using parsimonious flux balance analysis (pFBA) optimizing for maximum yield of mouse biomass objective function as defined in iMM1865 and minimum sum of flux (pFBA) 40. The ratio of metabolite abundance was calculated based on 20 hrs *vs.* 0 hrs data for *Hmox1*^*fl/fl*^ and *Hmox1*^*R26Δ/Δ*^. Next, the feasible flux values of exchange reactions for measured metabolites were modified by multiplying the flux of the given pFBA solution with calculated metabolite ratios for 20 hrs *vs.* 0 hrs for *Hmox1*^*fl/fl*^) and *Hmox1*^*R26Δ/Δ*^ individually resulting in two parameterized models iMM1865hmox and iMM1865hcre, respectively. If no exchange reactions were present for a measured metabolite, artificial exchange reactions were added to enable simulation of metabolite consumption or secretion. Next, flux variability analysis (FVA)^49^ was performed for both iMM1865hmox and iMM1865hcre. FVA was calculated as implemented in COBRApy seeking loopless solutions that adhere to 0.9 of the optimal biomass objective function value and at most 10% more than the minimum necessary sum of overall flux (flux_variability_analysis function with fraction_of_optimum=0.9, loopless=True and pfba_factor=1.1 setting). Loopless solution is calculated according to.^87^ Simulation results were further analyzed using R (v3.6.3) and tidyverse (v1.3.0). Differences in *Hmox1*^*fl/fl*^ and *Hmox1*^*R26Δ/Δ*^ conditions were determined by comparing feasible flux ranges per reaction. A reaction was identified to show altered flux if either lower or upper feasible reaction flux bound was different.

#### *In vitro* experiments

##### Cell culture rescue experiments

Cells were preincubated with 10 μM Dehydroascorbic acid (DHA) dissolved in DMEM+Penicillin-Streptomycin, without FBS for one hour. Then 20 μM hemin were added for additional 22 hours. Cell survival was determined with the XTT Cell Proliferation Assay (Serva) in a Spark® Multimode Microplate Reader (Tecan) according to manufacturer’s instructions.

##### Determination of G6PD and 6PGD enzymatic activity

Cell culture experiments were performed using mouse proximal tubule (RPTEC) cells and RPTEC *Hmox1* knockout (*Hmox1*^-/-^) cultured in Dulbecco’s modified Eagle’s medium (DMEM, Pan Biotech #P04-01550, Aidenbach, Germany), supplemented at 10 % Fetal Bovine Serum (FBS, Gibco #10270106, New York, NY, USA) and 1 % Penicillin-Streptomycin (PS, Gibco #15140122, New York, NY, USA). All cultures were incubated in a humidified atmosphere with 5 % CO_2_ at 37° C.

To assess the influence of *Hmox1* on the activation of the Pentose Phosphate Pathway (PPP) upon heme stimulation, 300,000 cells/well were seeded in 6-well plates and were allowed to grow for 24 hrs. Then, cells were washed with PBS and fresh media supplemented with either PBS or 50 μM heme, was added to the control and stimulated groups, respectively. 24 hrs after stimulation, cells were washed with PBS and harvested using trypsin (Trypsin, Pan Biotech #P10-0235SP, Aidenbach, Germany) in a humidified atmosphere with 5% CO_2_ at 37 °C for 5 minutes, followed by centrifugation at 2000 g for 5 min. Pellets were resuspended in PBS, 2x10^6^ cells were transferred to 2 mL tubes and centrifuged again to remove any traces of trypsin or culture media. Finally, the pellets were resuspended in 100 μL of cold PBS and cells were homogenized by quick pipetting followed by 10 min incubation at 0°C. Samples were centrifuged for 10 min. at 4 °C at 12,000 g to remove any insoluble material and supernatant were transferred to new tubes. Alternatively, kidney from *Hmox1*^*fl/fl*^ or *Hmox1*^*R26Δ/Δ*^ mice subjected or not to hemin were used. The protein concentration was determined for each sample using a BCA Protein Assay Macro Kit (BCA, SERVA #39228.02, Heidelberg Germany) and sample concentration was homogenized with PBS buffer. Finally, the enzymatic activity of glucose-6-phosphate dehydrogenase (G6PD) and 6-phosphogluconate dehydrogenase (6PGD) were determined using a Glucose-6-phosphate Dehydrogenase Assay kit (Abcam #ab102529, Cambridge, UK) and the 6-phosphogluconate Dehydrogenase Assay Kit (Abcam #ab241016, Cambridge, UK) following the manufacturer’s standard procedures. All experiments were done by triplicated.

### Quantification and statistical analysis

Data are presented as mean ± SD when appropriate for independent experiments as indicated in each figure legend. Statistical analysis was performed using GraphPad Prism version 9.0.0 (GraphPad Software Inc., USA) and significance was accepted when p value was below 0.05. Survival Plots were analyzed using the Log-rank test. Unpaired two-tailed Student’s t-test was performed when two independent groups were compared. When more than two groups were compared, One-way ANOVA or Two-way ANOVA with Tukey’s or Šidák’s *post hoc* test was performed as appropriate. Details are also provided in the respective figure legends.

## Supplemental information

Document S1. Figures S1–S9

Table S1. Dseq2 file Transcriptomics
