# Supplementary material for: Control of renal central carbon metabolism by heme oxygenase-1
Source: iScience. 2025 Dec 6;29(1):114370. doi: 10.1016/j.isci.2025.114370 (PMC12803938; doi:10.1016/j.isci.2025.114370)
Supplement: Document S1. Figures S1–S9 [file mmc1.pdf]

iScience, Volume *29*

Supplemental information

Control of renal central carbon metabolism by heme oxygenase-1

Joel Guerra, Elisa Jentho, Sascha Schäuble, Brendon P. Scicluna, Wolfgang Vivas, Dania Martínez-Alarcón, Marco Groth, Emma King, Franziska Röstel, Gianna Hirth, Miguel P. Soares, Thorsten Wiech, Gianni Panagiotou, Michael Bauer, Verena Hoerr, and Sebastian Weis

CONTROL OF RENAL CENTRAL CARBON METABOLISM BY HEME OXYGENASE-1

-SUPPLEMENTAL FIGURES-

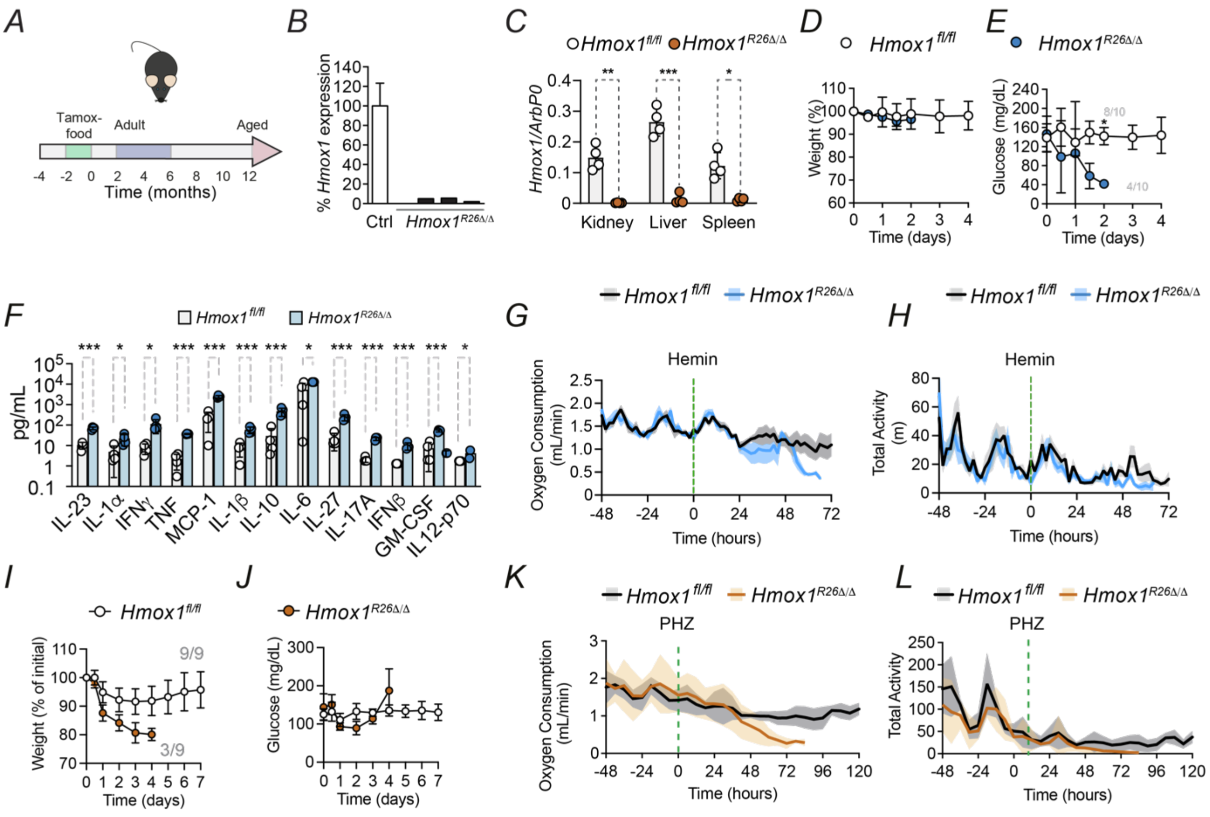

Supplemental Figure 1. HO-1 expression supports energy expenditure after heme injection and during intravascular hemolysis

(**A**) Experimental set-up. (**B**) qRT-PCR from whole blood leukocytes used for deletion assessment of *Hmox1*. Data are shown as mean+SD from two *Hmox1*$^{fl/fl}$ (Ctrl) and individual *Hmox1*$^{R26\Delta/\Delta}$ animals. (**C**) qRT-PCR from different organs 72 hrs after phenylhydrazine application. (**D**) weight and (**E**) glucose of heme treated *Hmox1*$^{fl/fl}$ *and Hmox1*$^{R26\Delta/\Delta}$ mice. (**F**) Peritoneal cytokines measured by Legendplex™ 12 hrs after heme application. (**G**) Oxygen consumption rate and (**H**) activity starting 48 hrs before heme injection (dashed line). (**I**) weight and (**J**) glucose of heme treated *Hmox1*$^{fl/fl}$ *and Hmox1*$^{R26\Delta/\Delta}$ mice. (**K**) Oxygen consumption rate and (**L**) activity starting 48 hrs before phenylhydrazine (PHZ) application (dashed line). *p<0.05; **p<0.01; ***p<0.001.

<u>Abbreviations:</u> Ctrl.. control; hrs.. hours

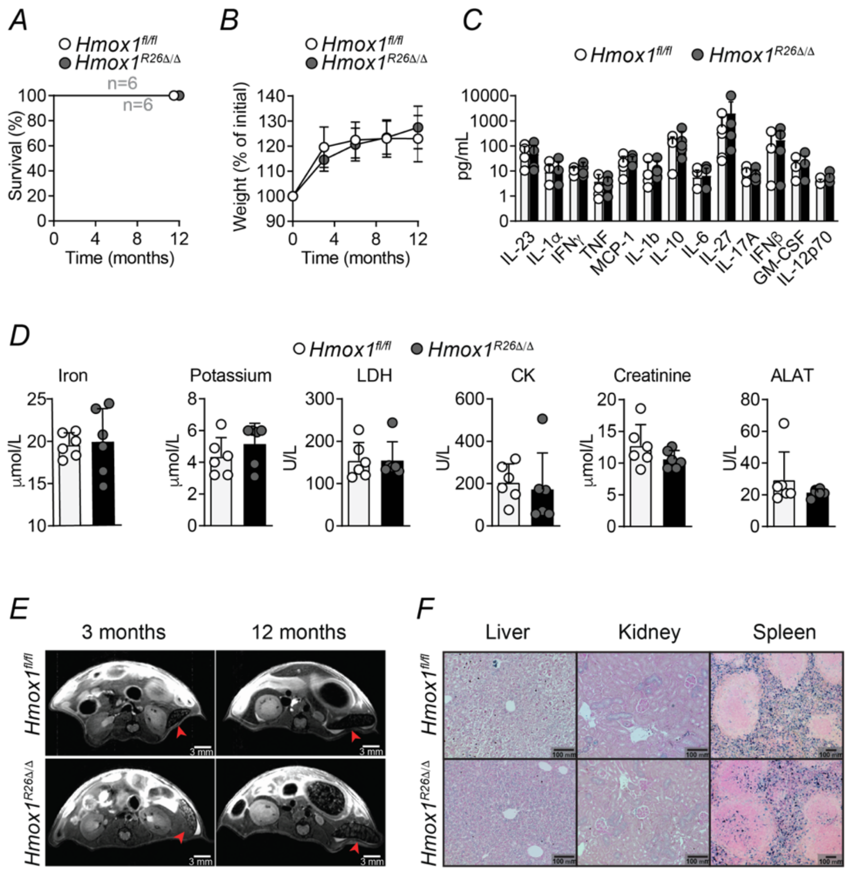

Supplemental Figure 2. Conditional deletion of *Hmox1* does not impair vigor
(**A**) Survival and (**B**) relative body weight of *Hmox1*$^{R26\Delta/\Delta}$ and *Hmox1*$^{fl/fl}$ over 12 months after *Hmox1*. Pooled from 2 experiments. (**C**) Plasma cytokines measured by Legendplex™ and (**D**) organ damage markers 12 months after deletion in *Hmox1*$^{R26\Delta/\Delta}$ and *Hmox1*$^{fl/fl}$. Dots represent individual animals. (**E**) Representative MRI of the abdomen in axial view at 3 and 12 months after *Hmox1* deletion or the same animals. Red arrows point to spleen. (**F**) Prussian blue staining of liver, kidney and spleen. All data is presented as mean ± SD. Dots indicate individual animals.

<u>Abbreviations:</u> ALAT.. alanine-transferase; Ctrl.. control; CK..creatine kinase; LDH.. lactate dehydrogenase.

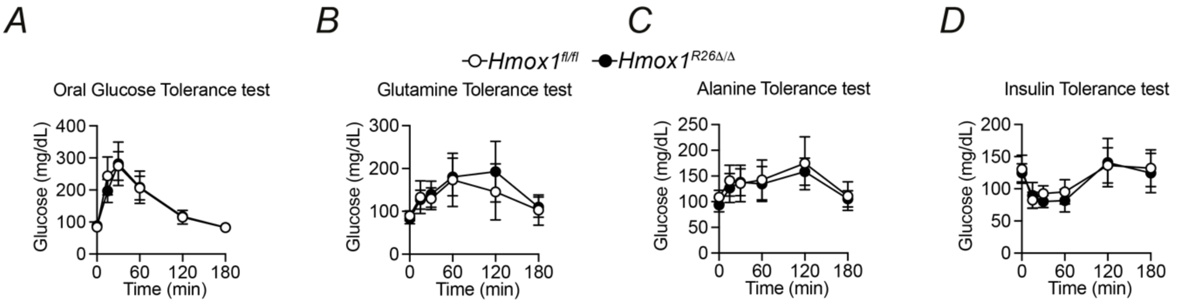

Supplemental Figure 3. Conditional *Hmox1* deletion does not impair glucose, glutamine, alanine or insulin metabolism at baseline

Tolerance tests to *Hmox1*$^{fl/fl}$ *and Hmox1*$^{R26\Delta/\Delta}$. (**A**) Glucose tolerance test. (**B**) Glutamine tolerance test. (**C**) Alanine tolerance test. (**D**) Insulin tolerance test. Data is presented as mean ± SD from 2 independent experiments, n= 6 animals per group.

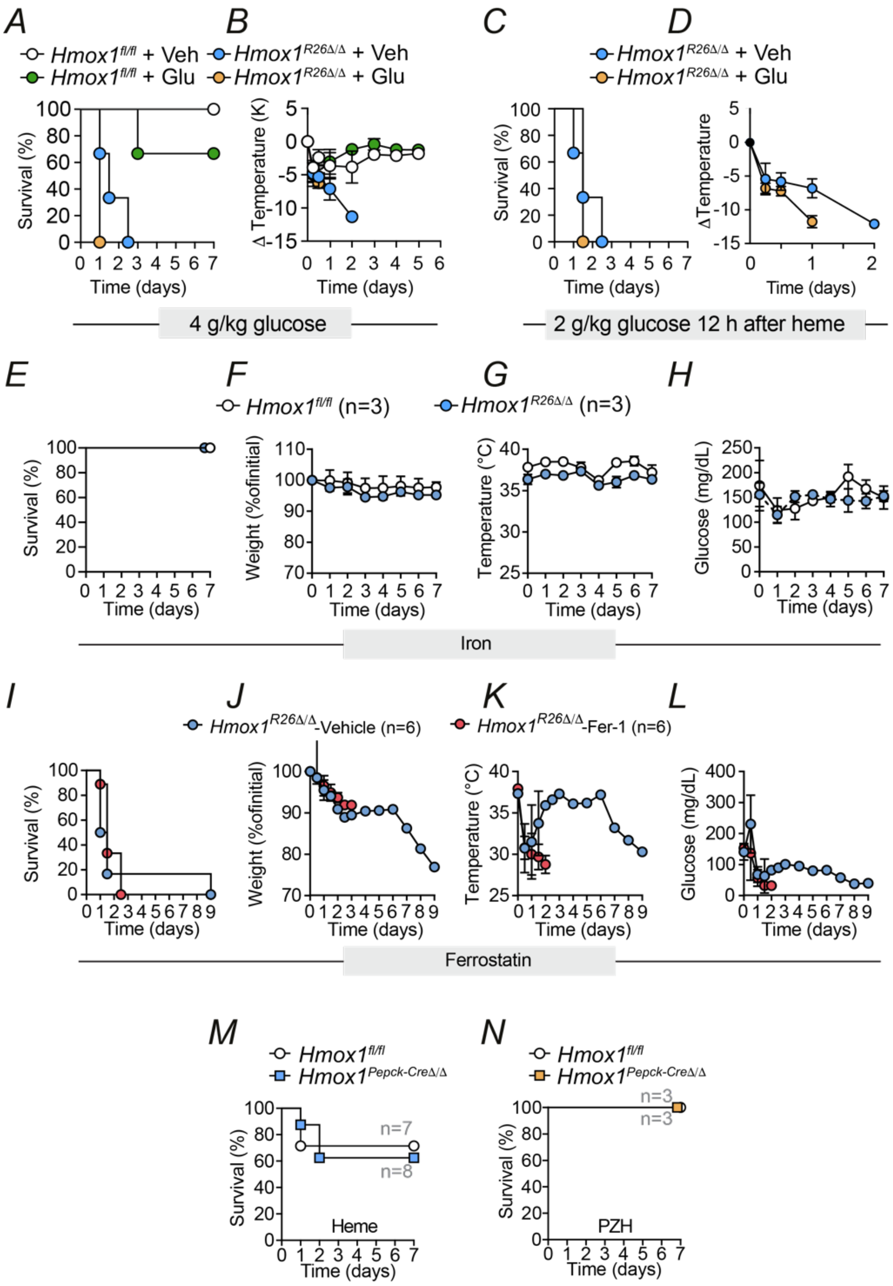

Supplemental Figure 4 (Related to **Fig. 2**)

(**A**) Survival and (**B**) relative temperature of heme-treated mice that received 4 g/kg glucose twice daily, n=3 mice/group from one experiment. (**C**) Survival and (**D**) relative temperature of heme-treated mice that received 2 g/kg glucose starting 12 hrs after heme application twice daily, n=3 mice/group from one experiment. (**E**) Survival, (**F**) relative weight, (**G**) temperature and (**H**) blood glucose of $Hmox1^{fl/fl}$ (Ctrl) and $Hmox1^{R26\Delta/\Delta}$ animals that received iron i.p., n=3 mice/group from one experiment. (**I**) Survival, (**J**) relative weight, (**K**) temperature and (**L**) blood glucose of $Hmox1^{R26\Delta/\Delta}$ animals that received vehicle or ferrostatin-1, n=6 mice/group from two independant experiments. (**M**, **N**) Survival of $Hmox1^{fl/fl}$ and $Hmox1^{Pepck.Cre\Delta/\Delta}$ mice after (**E**) heme (data from two independent experiments) and (**F**) phenylhydrazin injection (data from one experiment).

Abbreviations: Glu.. Glucose K.. Kelvin Veh: vehicle.

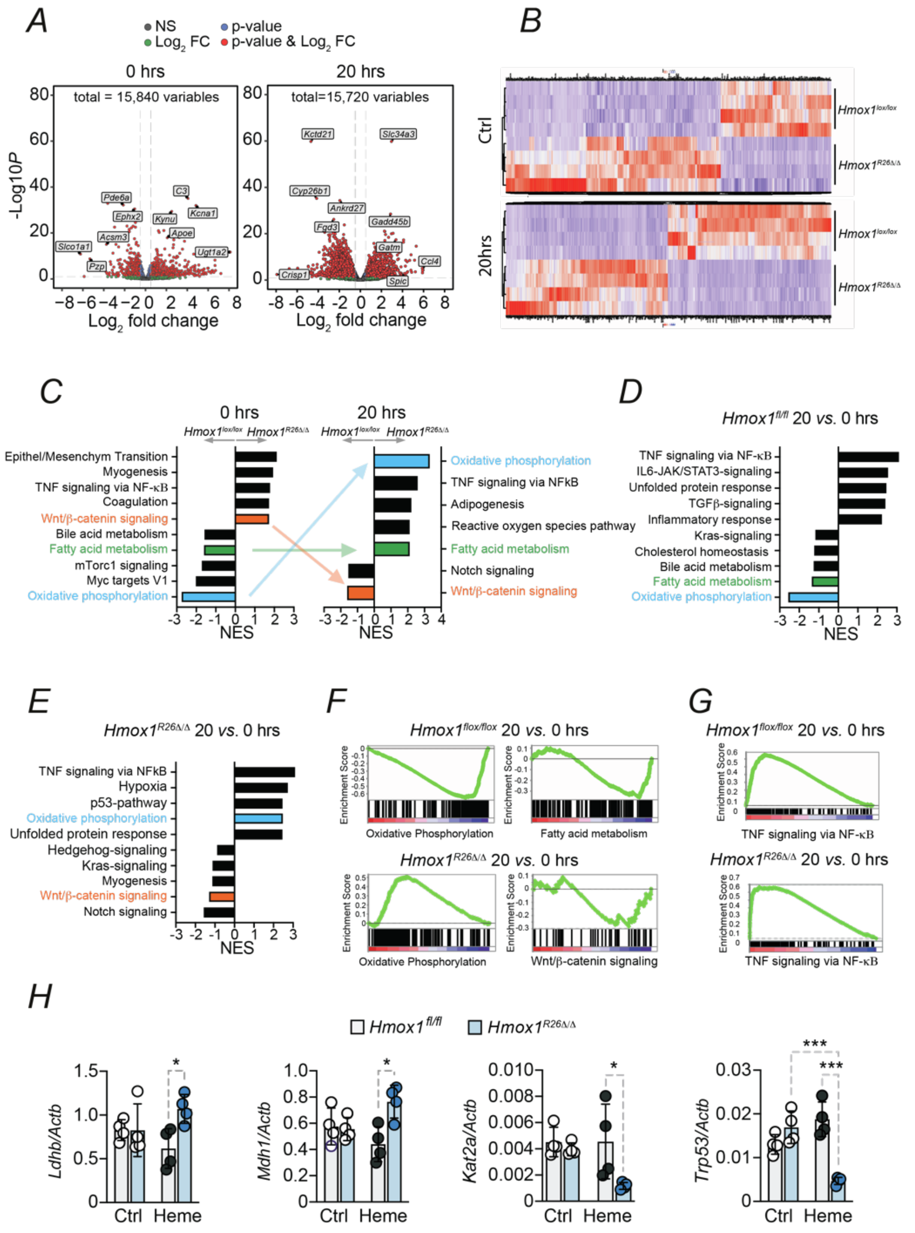

Supplemental Figure 5. Kidney transcriptome in *Hmox1*$^{fl/fl}$ and *Hmox1*$^{R26\Delta/\Delta}$ (Related to **Fig. 3**). Animals at baseline (0 hours) and 20 hrs after heme application. (**A**) Volcano plots, (**B**) heat maps and (**C**) ranked significantly (FDR < 0.05) enriched pathways on hallmark biological processes using normalized enrichment score (NES) between *Hmox1*$^{fl/fl}$ and *Hmox1*$^{R26\Delta/\Delta}$ animals at indicated time points. (**D,E**) Ranked NES of hallmark biological processes (**D**) *Hmox1*$^{fl/fl}$ and (**E**) *Hmox1*$^{R26\Delta/\Delta}$ animals from time series analysis including RNAseq data at baseline and 20 hrs after heme application. (**F, G**) GSEA of selected hallmark pathways (**F**) differently and (**G**) similarly regulated in *Hmox1*$^{fl/fl}$ and *Hmox1*$^{R26\Delta/\Delta}$ animals over time. (**H**) qRT-PCR of up- and down-regulated genes. Data is derived from kidney of animals treated or not with heme for 12 hours and shown as mean + SD. Each dot represents an individual animal. Two-way ANOVA with Šidák correction. *p<0.05; ***p<0.001.

Abbreviations: FC.. fold change; hrs.. hours; NES.. normalized enrichment score.

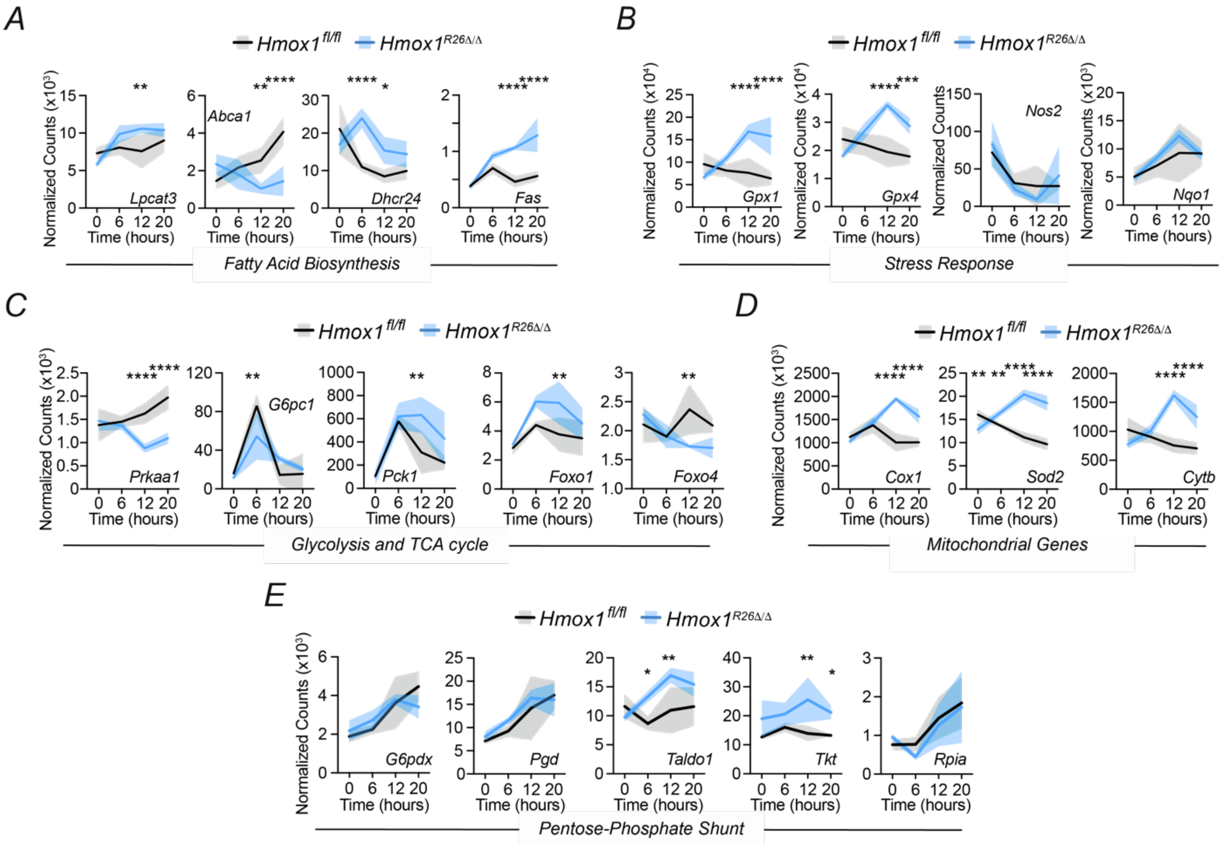

Supplemental Figure 6 Heme-induced transcriptional modifications in murine kidneys (Related to **Fig. 3**)

Time-resolved mRNA expression of genes regulating (**A**) lipid biogenesis or (**B**) stress response pathways. (**C**) energy metabolism, (**D**) mitochondrial function and (**E**) Pentose phosphate pathway. Extracted data from RNAseq analysis and shown as mean + SD from 3-4 animals per group. Two-way ANOVA with Šidák correction. Data is derived from individual mice per time point but displayed with a connecting line for better visualization. *p<0.05; **p<0.01; ***p<0.001; ****p<0.0001.

<u>Abbreviations:</u> *Abca1*.. ATP binding cassette subfamily A member 1; *Cox1*.. Cyclooxygenase-1; *Cytb*.. cytochrome b; *Dhcr24*.. 24-Dehydrocholesterol Reductase; *Fas*.. fas cell surface death receptor; *Foxo1*.. forkhead box O1; *Foxo4*.. forkhead box O4; *Gpx1*.. glutathione peroxidase 1; *Gpx4*.. glutathione peroxidase 4; *G6pc*.. Glucose-6-phospatase, catalytic subunit; *G6pdx*.. glucose-6-phosphate dehydrogenase X-linked; *Lpcat3*.. lysophosphatidylcholine acyltransferase 3; *Nos2*.. nitric oxide synthase 2; *Nqo1*.. NAD(P)H quinone dehydrogenase 1; *Pck1*.. phosphoenolpyruvate carboxykinase 1; *Pgd*.. phosphogluconate dehydrogenase; *Prkaa1*.. catalytic subunit of protein kinase, AMP-activated, alpha 1; *Rpia*.. ribosome 5-phosphate isomerase A; *Sod2*.. superoxide dismutase-2; *Taldo1*.. transaldolase-1; *Tkt*.. transketolase.

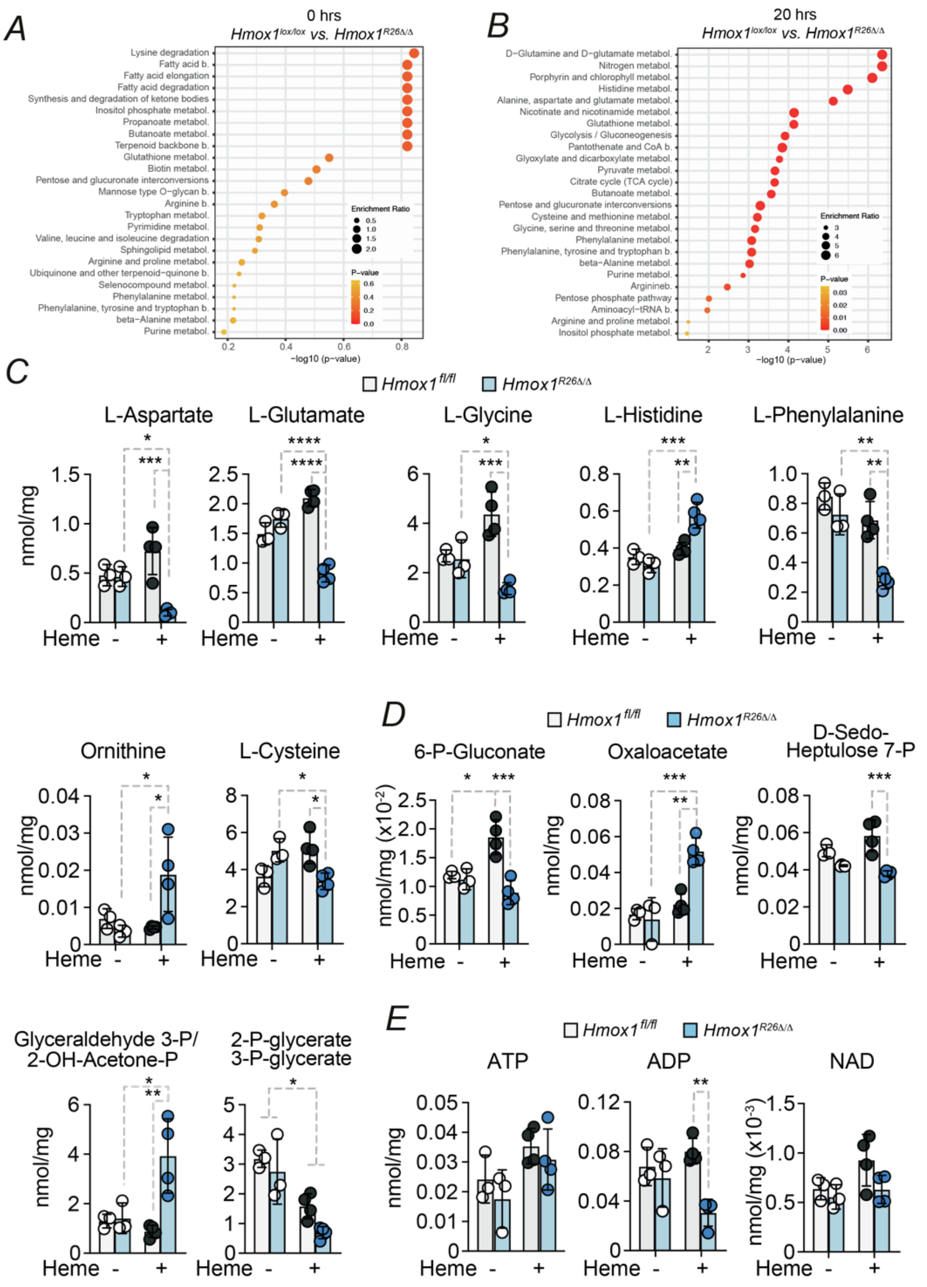

Supplemental Figure 7. Targeted metabolomics of the kidney (Related to **Fig. 4**)

Enriched metabolic pathways in kidney (***A***) without heme and (***B***) 20 hrs after heme application. (***C***) amino acids, (***D***) small molecules and (***E***) glycolysis/TCA-cycle metabolites. Data are shown as mean ± SD of 3-4 mice per group. Dots indicate individual animals. Two-Way ANOVA with Šidák correction. *p<0.05; **p<0.01; ***p<0.001; ****p<0.0001.

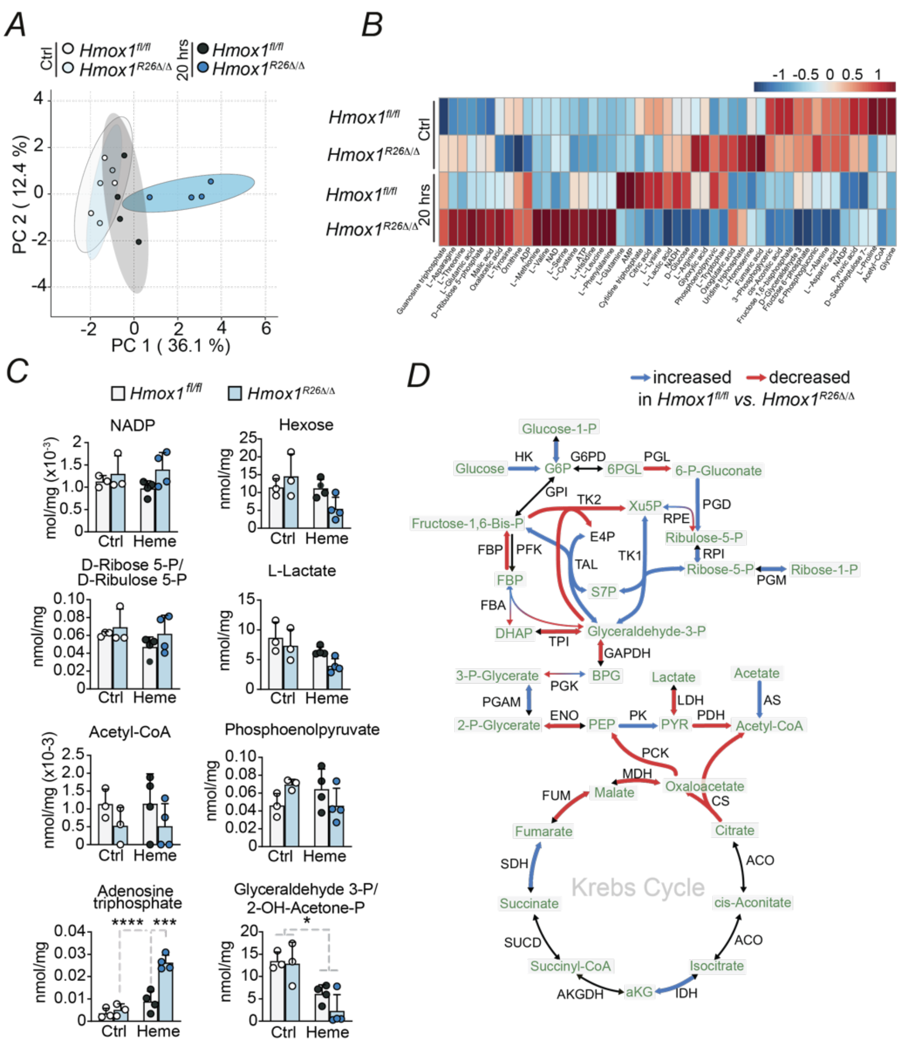

Supplemental Figure 8 Integrated hepatic metabolomics (Related to **Fig. 4**)

(**A**) Principal Component Analysis (PCA) for liver metabolites**.** Data show difference between 14 samples of untreated and 20 hrs heme-injected animals (4 different conditions with each with n= 3 to 4 animals per group). (**B**) Heatmap analysis highlighting metabolites after normalization of non-stimulated or 20 hrs heme stimulated animals. n= 3 to 4 animals per group. (**C**) Selected metabolites of the liver and (**D**) Targeted flux balance analysis for ATP generation using data integration into a reconstructed metabolic network. Data are shown as mean ± SD (n=3-4 mice per group). Dots represent individual animals. Two-Way ANOVA with Šidák correction. *p<0.05; ***p<0.001; ****p<0.0001.

Abbreviations; AC.. Acetate; ACCOA.. Acetyl-CoA; ACON.. Aconitate; AKG.. α-Ketoglutarate; BPG.. 1;3-Bisphosphoglycerate; CIT.. Citrate; DHAP.. Dihydroxyacetone phosphate; E4P.. Erythrose 4-phosphate; FDP.. Fructose 1;6-bisphosphate; FUM.. Fumarate; F6P.. Fructose 6-phosphate; G1P.. Glucose 1-phosphate; G6P.. Glucose 6-phosphate; G3P.. Glyceraldehyde 3-phosphate; ICIT.. Isocitrate; LAC.. Lactate; MAL.. Malate; OAA.. Oxaloacetate; PEP.. Phosphoenolpyruvate; PYR.. Pyruvate; Ru5P.. Ribulose 5-phosphate; R1P.. Ribose 1-phosphate; R5P.. Ribose 5-phosphate; SUCC.. Succinate; SUCCOA.. Succinyl-CoA; S7P.. Sedoheptulose 7-phosphate; XuP5.. Xylulose 5-phosphate; 2PG.. 2-Phosphoglycerate; 3PG.. 3-Phosphoglycerate; 6PGC.. 6-Phosphogluconate; 6PGL: 6-Phosphogluconolactone.

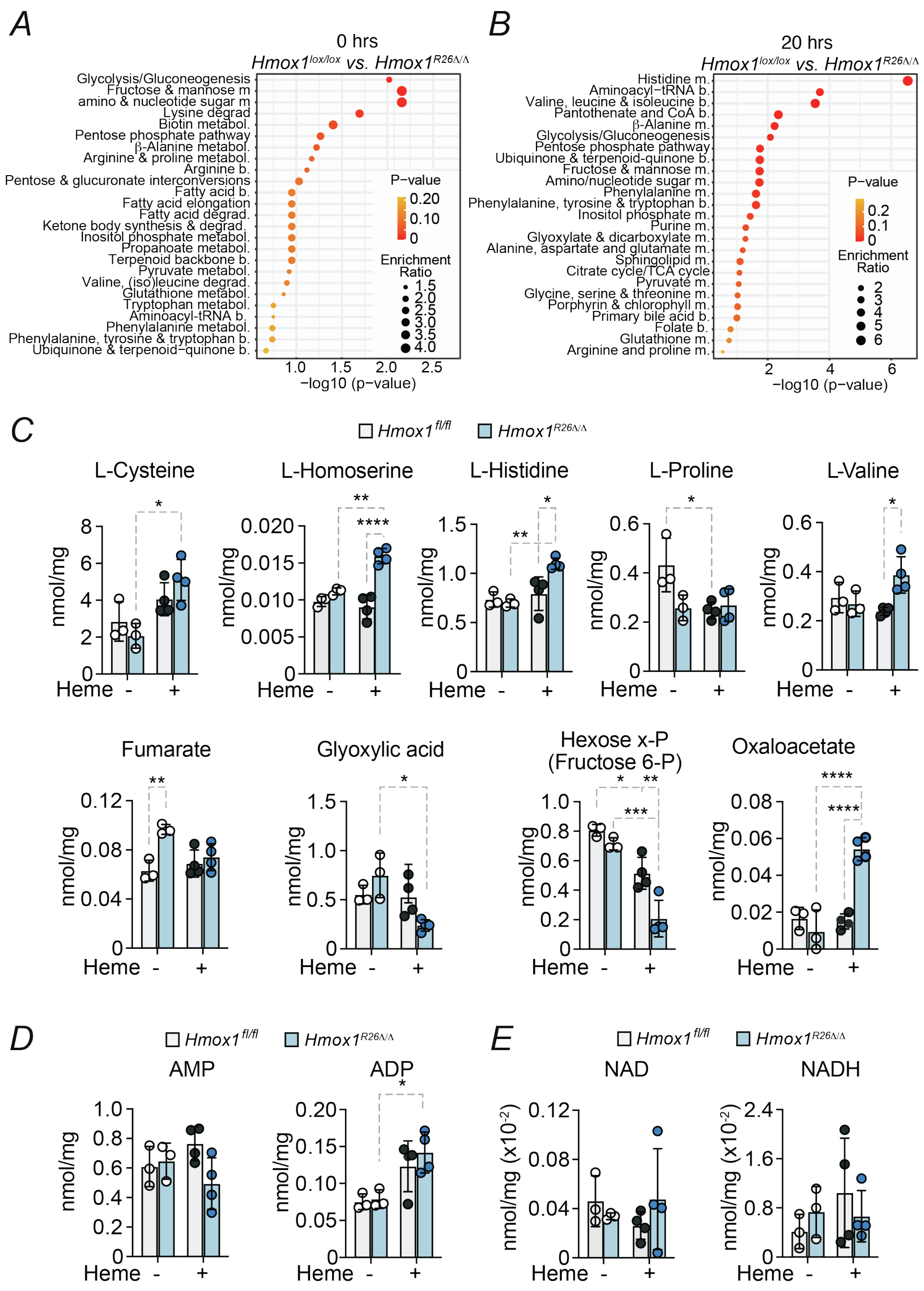

Supplemental Figure 9 Targeted metabolomics of the liver (Related to **Fig. 4**)

Enriched metabolic pathways in liver (***A***) without heme and (***B***) 20 hrs after heme application. Significantly changed (***C***) amino acids, (***D***) small molecules and (***E***) glycolysis/TCA-cycle metabolites. Data is shown as mean ± SD of 3-4 mice per group. Dots indicate individual animals. Two-Way ANOVA with Šidák correction. *p<0.05; **p<0.01; ***p<0.001.; ****p<0.0001.

<u>Abbreviations</u>: b.. biosynthesis; m.. metabolism
